# ERβ-Score: An Interpretable Machine Learning-Based Scoring Function and Web Server for Estrogen Receptor β-Guided Drug Discovery in Triple-Negative Breast Cancer

**DOI:** 10.3390/ijms27167089

**Published:** 2026-08-07

**Authors:** Abbas Khan, Muhammad Ammar Zahid, Walid Kouidri, Osama Aboubakr Mohamed, Ahmed Mohammad Gharaibeh, Ladun Ibrahim Mohamed, Amani Anwar Al-Mansori, Mohamed Haitham Elsayed, Anwar Mohammad, Ameera Al-Jabiry, Mohanad Shkoor, Raed M. Al-Zoubi, Abdelali Agouni

**Affiliations:** 1 Department of Pharmaceutical Sciences, College of Pharmacy, QU Health, Qatar University, Doha P.O. Box 2713, Qatar; abbas.khan@qu.edu.qa (A.K.); mz1912625@qu.edu.qa (M.A.Z.); wk1902655@qu.edu.qa (W.K.); oa2003649@qu.edu.qa (O.A.M.); ag2107297@qu.edu.qa (A.M.G.); lm2006178@qu.edu.qa (L.I.M.); aa2004409@qu.edu.qa (A.A.A.-M.); me1901975@qu.edu.qa (M.H.E.); 2 Precision Health Analysis Unit, Translational Research, Dasman Diabetes Institute, Dasman 15462, Kuwait; anwar.mohammad@dasmaninstitute.org; 3 Department of Chemistry and Earth Sciences, College of Arts and Science, Qatar University, Doha P.O. Box 2713, Qatar; a.aljabiry@qu.edu.qa (A.A.-J.); mshkoor@qu.edu.qa (M.S.); 4 Surgical Research Section, Department of Surgery, Hamad Medical Corporation, Doha P.O. Box 3050, Qatar; 5 Department of Biomedical Sciences, College of Health Sciences, QU Health, Qatar University, Doha P.O. Box 2713, Qatar; 6 Department of Chemistry, Jordan University of Science and Technology, P.O. Box 3030, Irbid 22110, Jordan

**Keywords:** ERβ, machine learning, Y-randomization, virtual screening, web application, breast cancer

## Abstract

Triple-negative breast cancer (TNBC) is the most clinically aggressive subtype of breast cancer, characterized by the absence of targetable hormone receptors and HER2 amplification, significantly constraining treatment choices. Estrogen Receptor Beta (ERβ) has emerged as a biologically relevant yet underutilized target in TNBC, with its re-expression linked to tumor suppression and improved prognosis, prompting the development of selective ERβ modulators as a precision therapeutic approach. We introduce ERβ-Score, an interpretable machine learning scoring system developed using a curated dataset of 1699 ERβ bioactive chemicals obtained from ChEMBL, characterized by 39 physicochemical and three-dimensional molecular descriptors. After implementing scaffold-disjoint train/test partitioning to avert structural data leakage, a Gradient Boosting Classifier, fine-tuned through Bayesian hyperparameter optimization, attained in five-fold cross-validation a Precision–Recall AUC (Area Under the Curve) of 0.891, a ROC-AUC (Receiver Operating Characteristic) of 0.888, a Matthews Correlation Coefficient of 0.664, an F1-score of 0.838, and a balanced accuracy of 0.831; on the scaffold-disjoint hold-out test set it attained a Precision–Recall AUC of 0.905, a ROC-AUC of 0.864, and a Matthews Correlation Coefficient of 0.578, indicating strong and balanced discrimination between active and inactive ERβ modulators. We note explicitly that this scaffold-disjoint hold-out constitutes internal validation, since it derives from the same curated ChEMBL workflow used for model development, and it is therefore reported throughout as scaffold-disjoint internal validation rather than as independent external validation. The applicability domain boundaries were established using a k-nearest-neighbor Tanimoto-similarity method with ECFP4 (Extended-Connectivity Fingerprint with a Diameter of 4) fingerprints, offering a quantitative confidence metric that identifies structurally new molecules beyond the model’s reliable prediction range. External validation against independent Tox21 ERβ bioassay data confirmed genuine, statistically significant predictive signal (ROC-AUC = 0.71) while revealing reduced sensitivity for structurally novel active compounds. The model was subsequently used for extensive virtual screening of natural product and drug-like compound libraries, with prioritized candidates undergoing structure-based molecular docking against the ERβ co-crystal structure (PDB: 7XWQ) using Smina, facilitating a comprehensive evaluation of hits based on both ligand and structural properties. To enhance accessibility, the complete pipeline was implemented as an open-access interactive web application utilizing Streamlit, enabling researchers to input any SMILES string and obtain, in real time, an activity prediction with a probability score, applicability domain classification, Lipinski drug-likeness assessment, interactive three-dimensional visualization of protein–ligand interactions, and on-demand docking within the ERβ active site.

## 1. Introduction

Breast cancer is the most prevalent malignancy among women and the leading cause of death from cancer in females worldwide [1,2]. Nationally, according to data from the Qatar National Cancer Registry of 2020, breast cancer was the most common cancer in Qatar (218 cases or 14.7% of new cases) when both sexes are considered. Among women, breast cancer is the most common cancer, representing 37.7% of all female cancer cases. It is ranked 3rd as a cause of cancer mortality in Qatar (57 deaths or 8.1%) when both sexes are considered. Among breast cancer subtypes, the estrogen receptor (ER)-positive luminal α and β subtypes are the most common form (70% of all cases). The overexpression of ER supports fast proliferation and survival of breast cancer cells [3,4,5,6]. Tumors from about 50% of ER-positive breast cancer patients contain ERα alone, 10% express ERβ without ERα, and the rest express both ER subtypes. ERα is believed to be the main driver of its tumorigenic effects; however, ERβ may inhibit ERα-mediated transcriptional responses [5,7,8,9]. ERβ exhibits tumor-suppressor activity in several biological contexts. For instance, ERβ suppression in ERα-positive cell lines led to the development of an invasive phenotype and enhanced cell proliferation [10]. When estradiol is present, ERβ overexpression decreases cell proliferation and inhibits tumor growth [11,12].

Therefore, targeting the ERβ receptor with selective agonists in breast cancer treatment may offer benefits by regulating its function to inhibit tumor growth, especially when the ERα is overly active or unresponsive to therapy. This approach has the potential to provide a more customized and efficient treatment plan for certain subtypes of breast cancer. However, the lack of ERβ-selective agonists that target ERβ without affecting ERα has hindered the investigation of ERβ activation as a potential approach for treating ER-positive breast cancer. Therefore, this project aims to design novel ERβ agonists for the treatment of hormone-positive breast cancer. ERα is associated with good clinical outcomes when treated with endocrine therapy, while targeting the ERβ subtype presents diagnostic and therapeutic challenges [13]. Tamoxifen, Toremifene, and Fulvestrant are drug classes with activity at the ER. Tamoxifen and toremifene are known as selective ER modulators (SERMs); they have mixed agonist and ER Degrader (ERD) activities [14,15,16,17,18]. They bind to the ER and block estrogen’s action in breast tissue. However, they have estrogen-like effects in other tissues, which can cause side effects such as hot flashes, sweating, and muscle and joint aches. In contrast, fulvestrant is a pure ERD that binds the ER and promotes its degradation, thereby blocking all ER activity. Fulvestrant is also without side effects caused by ER-agonist action in non-breast tissues [19,20,21,22].

Artificial Intelligence is transforming drug discovery by changing how therapeutic candidates are identified and developed, in ways that differ substantially from conventional approaches. Traditionally, drug discovery relied on high-throughput screening of sequentially synthesized compounds, which is limited in its ability to explore the vast number of possible chemical entities (~10^60^). In contrast, recent advances in machine learning enable the extraction of complex, non-linear structure–activity relationships from large bioactivity datasets, supporting a shift from trial-and-error approaches toward more predictive and data-driven strategies. Advanced ensemble methods, particularly gradient boosting algorithms, can capture subtle molecular patterns that are often inaccessible to conventional techniques. Recent applications in kinase inhibitor design, G-protein-coupled receptors (GPCRs), and nuclear receptor modulation illustrate the growing role of AI in virtual screening, enabling the prioritization of millions of compounds in a short time. As a result, AI is reshaping early-stage drug discovery by improving efficiency, reducing cost, and enhancing predictive accuracy, and is increasingly becoming a key driver in the development of next-generation therapeutics.

In this study, we develop ERβ-Score, an interpretable machine-learning-based scoring framework to predict the probability of compound activity against ERβ, enabling precision drug discovery in breast cancer. The model integrates robust descriptor engineering, scaffold-aware data splitting, and Bayesian hyperparameter optimization to ensure generalizable performance across diverse chemical scaffolds. Model reliability is rigorously evaluated using Y-randomization and applicability domain (AD) analysis to distinguish confident predictions from extrapolations. The framework is further designed for practical deployment via model serialization, facilitating reproducible, scalable screening of large chemical libraries. Collectively, this work aims to provide a reliable computational tool for prioritizing ERβ modulators and supporting targeted therapeutic development.

## 2. Results and Discussion

### 2.1. Dataset Curation and Composition

**Figure 1:**
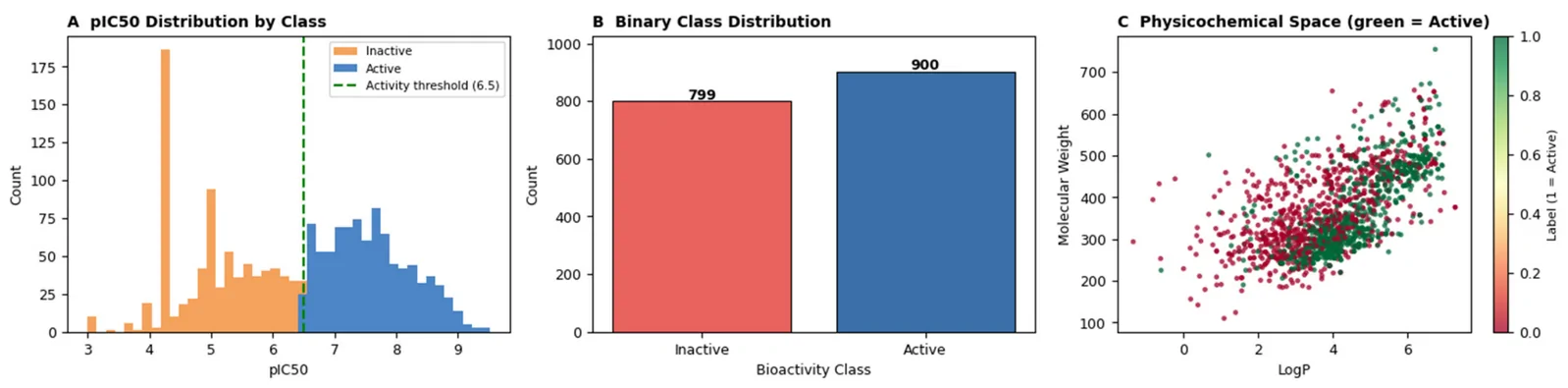
Data curation summary. (**A**) pIC_50_ distribution for the 1699 curated compounds with the single activity threshold (pIC_50_ = 6.5) marked; actives are shown in blue and inactives in orange. (**B**) Binary class distribution, showing 900 actives and 799 inactives. (**C**) Physicochemical property space coloured by bioactivity label, showing the distribution of the compounds by activity in relation to molecular weight and LogP.

The initial dataset retrieved from ChEMBL contained 3653 bioactivity records across 48 fields, which were subjected to a sequential curation process to ensure data quality and consistency. Removing the other bioactivity units and retaining only entries with IC_50_ values reported in nM reduced the dataset to 2732 entries. Afterward, entries with missing records yielded only 2665 entries, while the others were excluded. Removal of duplicates from the dataset further reduced the total to 2440 compounds, eliminating 225 duplicates. To improve reliability, compounds with inconsistent bioactivity measurements (standard deviation of pIC_50_ > 0.5) were excluded, eliminating 468 records corresponding to 190 compounds, and replicate measurements were consolidated, resulting in 1758 entries. Additional physicochemical filtering removed 59 compounds, yielding a final curated dataset of 1699 compounds. The pIC_50_ values ranged from 3.00 to 10.34. For classification purposes, all 1699 curated compounds were assigned a binary label using a single potency threshold, with compounds having pIC_50_ ≥ 6.5 labelled active (1) and those with pIC_50_ < 6.5 labelled inactive (0) (Figure 1A). This yielded 900 active and 799 inactive compounds (Figure 1B), a near-balanced distribution (53.0% active and 47.0% inactive), and no compounds were discarded at this step. We note for transparency that an exploratory dual-threshold scheme (pIC_50_ ≥ 7.5 active, pIC_50_ < 5.5 inactive) was examined during development; it retained 988 compounds (467 active, 521 inactive) and excluded 711 intermediate-activity compounds, but it was not adopted for model construction, and every result reported in this work derives from the single-threshold 1699-compound dataset defined above. These compounds were then displayed as a scatter plot based on their physicochemical properties, primarily molecular weight and AlogP (Figure 1C). The activity distribution is color-coded to understand the distribution and classify these compounds as weak, moderate, or strong, active or inactive. This balanced class distribution minimizes the need for aggressive resampling techniques and supports the use of PR-AUC as a reliable primary evaluation metric. The whole data summary is provided in Figure 1A–C.

### 2.2. Molecular Descriptors and Chemical Space Characterization

All 1699 SMILES were successfully parsed into valid RDKit Molecular Objects (Molecules) and textual representations, each with associated data. Four different types of fingerprints, such as ECFP4, ECFP6, MACCS, and RDKit FingerPrints, and fifty-four (54) different types of descriptors (44 × 2D and 10 × 3D) were also calculated for each Molecular Object. The ETKDG v3 produced three-dimensional conformers with a 100% success rate (0 embedding failures), suggesting that the curated dataset contains no topologically pathological structures. PCA of the ECFP4 fingerprint space showed partial, but not complete, separation of the active and inactive classes along the first two principal components, confirming that the classification task is non-trivially embedded in chemical space and requires multivariate modeling beyond simple linear separation. The overall distribution patterns of the 2D key descriptors, including MW, logP, TPSA, Hydrogen Bond Donor/Acceptor, showed overlapped but distinct profiles between both classes; specifically, the distributions of active compounds had a much greater average molecular weight, while still being relatively lipophilic by demonstrating higher logP, as compared to inactive compounds. Hence, these findings strongly support the evidence that the traditional Pharmacophore feature directly affects the activity of ERβ ligand-binding domain ligands. The 3D shape analysis of both classes, based on principal moments of inertia (PMI; NPR1 and NPR2), indicates that the two classes occupy similar conformational space, with substantial overlap in the spherical and disc-like regions. In contrast, rod-like shapes are minimally represented in both classes relative to the overall distribution (Supplementary Figure S1). The initial descriptor matrix contained 54 features with no missing values; therefore, no imputation was required. No zero-variance features were observed. However, three descriptors were removed using a near-zero-variance filter (variance < 0.01). To address redundancy, highly correlated features (|r| > 0.95) were identified, and one feature from each correlated pair was removed, resulting in a final dataset of 1699 compounds described by 39 features (Supplementary Figure S2).

### 2.3. Scaffold Analysis and Data Partitioning

**Figure 2:**
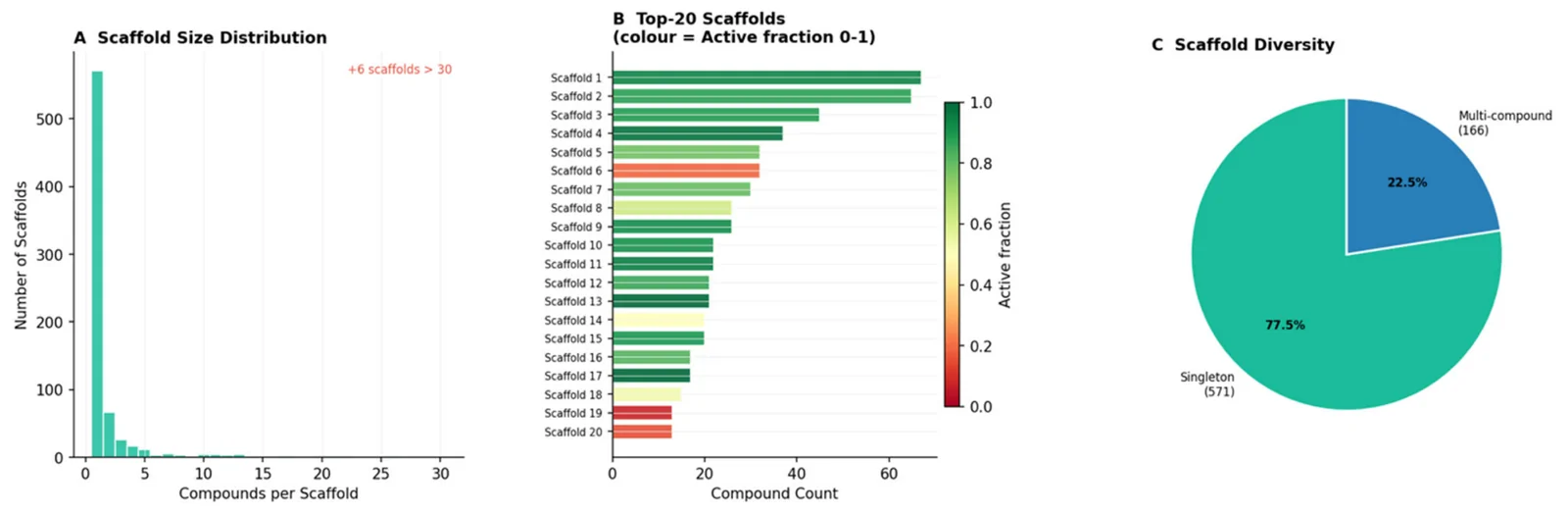
Scaffold-disjoint train/test split visualization. (**A**) Class distributions, (**B**) top 20 scaffolds colored on the active fraction (0–1), and (**C**) shows the scaffold diversity, whether singleton or multi-compound.

Bemis–Murcko scaffold analysis of the curated dataset (n = 1699) identified 737 unique scaffolds, corresponding to a scaffold diversity of 0.4338 (Figure 2A). Further analysis of the top 20 scaffolds (Figure 2B) reveals variability in activity distribution, with active fractions ranging from low to high, indicating that bioactivity is not confined to specific scaffold classes but is distributed across multiple chemotypes. This diversity is essential for developing robust machine learning models that capture generalized structure–activity relationships rather than overfitting to a limited set of dominant scaffolds. Overall, the high proportion of singleton scaffolds combined with moderate scaffold clustering underscores the suitability of this dataset for scaffold-disjoint validation, ensuring a realistic assessment of model performance on structurally novel compounds. The scaffold size distribution is highly skewed, with the majority of scaffolds represented by only a few compounds, as evidenced by 571 singleton scaffolds (77.5% of all unique scaffolds; Figure 2C). This indicates a chemically diverse dataset with limited redundancy, which is favorable for evaluating model generalization. The distribution of compounds per scaffold shows that most scaffolds contain fewer than 5 compounds, while only a small number are densely populated (>30 compounds), highlighting the presence of a few dominant chemotypes. Consistent with this, the top 10 scaffolds account for 382 compounds (22.5% of the dataset), suggesting moderate clustering within certain structural classes.

**Figure 3:**
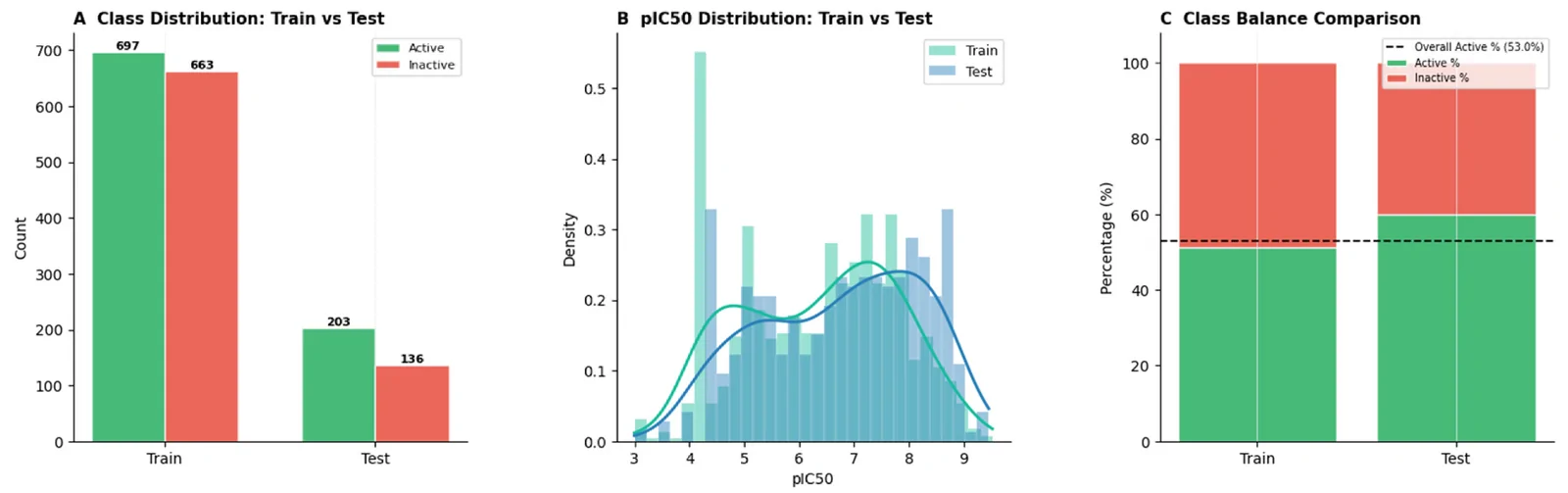
Scaffold-disjoint train–test split (80/20; SEED = 42). (**A**) Class distributions in the training (n = 1360) and test (n = 339) sets are nearly balanced. (**B**) pIC_50_ distributions are comparable across splits. (**C**) Slight enrichment of actives in the test set arises from scaffold-based partitioning, with no scaffold overlap ensuring unbiased generalization.

The scaffold-disjoint split (80/20) produced training (n = 1360) and test (n = 339) sets with no shared scaffolds, ensuring a strict evaluation of model generalization to unseen chemical space. As shown in Figure 3A, the class distribution is reasonably balanced across both partitions: the training set contains 697 active and 663 inactive compounds (51.2% active), while the test set contains 203 active and 136 inactive compounds (59.9% active). Although the test set shows a slightly higher proportion of active compounds, this variation arises from scaffold-based partitioning rather than sampling bias. The distribution of bioactivity values (pIC_50_) remains comparable between training and test sets (Figure 3B), indicating that both partitions cover a similar activity range (approximately 3–10 log units). The overlapping density profiles confirm that the model is trained and evaluated on chemically and biologically consistent spaces, despite scaffold separation. Further comparison of class balance shows that both sets maintain proportions close to the overall dataset distribution (53.0% active), with only a modest deviation in the test set (Figure 3C). This slight enrichment of active compounds does not artificially inflate performance, as PR-AUC used as the primary evaluation metric is sensitive to class prevalence and provides a conservative estimate under such conditions. Overall, the scaffold-disjoint strategy ensures no information leakage at the structural level, while preserving sufficient class balance and activity distribution across splits. This setup provides a realistic and rigorous framework for evaluating the model’s ability to generalize to novel scaffolds encountered in practical drug discovery scenarios.

### 2.4. ERβ-Score: Machine Learning Model Performance

#### 2.4.1. Cross-Validation Benchmark

Optuna HPO (20 TPE trials per algorithm) followed by full five-fold CV yielded a tightly clustered performance tier among the top models (Table 1). GradBoost achieved the highest mean CV PR-AUC of 0.8911 ± 0.021, followed by XGBoost (0.8905 ± 0.023), RandomForest (0.8898 ± 0.028), and ExtraTrees (0.8891 ± 0.025). The Δ13 between these four models is only 0.002 PR-AUC units, indicating that multiple tree-based algorithms converge to a similar decision surface for this descriptor set. All seven tree/boosting methods exceeded PR-AUC ≥ 0.8863, while MLP (0.8618), kNN (0.8514), and Logistic Regression (0.8195) showed comparatively lower performance, consistent with the expectation that tree-based ensembles are better suited to the heterogeneous, mixed-scale descriptor space used here. Notably, SVM-RBF achieved the highest MCC (0.6679) and balanced accuracy (0.8335) despite a marginally lower PR-AUC, reflecting its stronger calibration at the 0.5 decision threshold. The low standard deviations across folds (σ_PR-AUC_ < 0.030 for all models) confirm stable cross-validated performance and adequate fold sizes. The visual results are given in Supplementary Figure S3.

#### 2.4.2. Scaffold Hold-Out Test Set Performance

**Figure 4:**
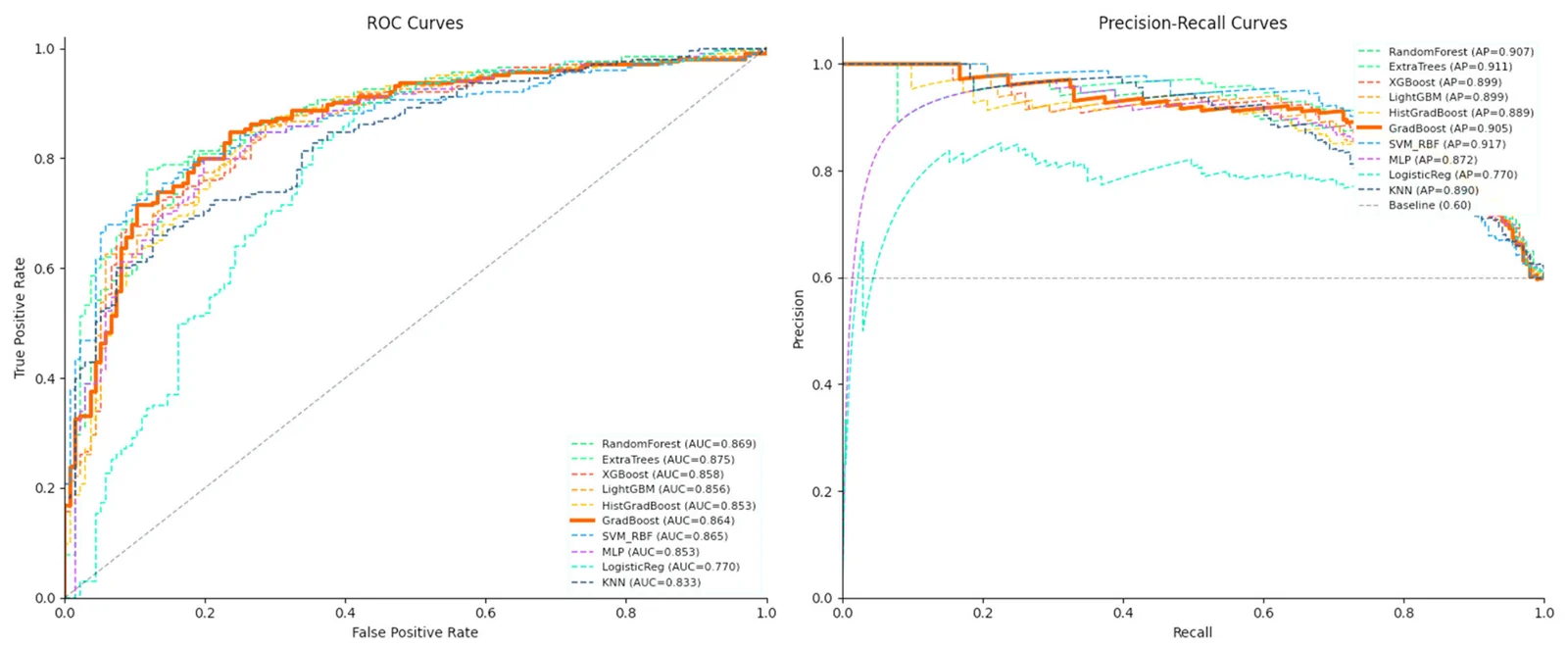
ROC and precision–recall curves for all 10 models on the scaffold hold-out test set. Gradient Boost (ERβ-Score) is shown as a bold solid line.

The performance of all models on the scaffold-disjoint test set is summarized in Table 2 and Figure 4. Across models, PR-AUC values remain consistently high (>0.89 for most ensemble methods), indicating strong ranking capability under a scaffold-shift scenario. Notably, SVM-RBF achieves the highest PR-AUC (0.9173), suggesting excellent ranking performance; however, its overall classification balance is slightly inferior to that of ensemble models, as indicated by MCC and balanced accuracy. The GradBoost model (ERβ-Score), retrained on the full 1360-compound training set, achieved a test-set PR-AUC of 0.9054 and ROC-AUC of 0.8638 on the 339-compound scaffold-disjoint hold-out set (MCC = 0.5779, F_1_ = 0.8053, balanced accuracy = 0.7947). The test PR-AUC (0.9054) exceeding the CV estimate (0.8911) is noteworthy and suggests that the test fold, while structurally novel, contains chemotypes whose SAR patterns align with learned training-set relationships, a favorable sign for prospective screening. The slightly lower MCC on the test set (0.5779 vs. 0.6637 CV) reflects the challenge of scaffold-disjoint prediction, where the model must extrapolate beyond known scaffolds. The ROC and precision–recall curves (Figure 4) further confirm that all tree-based ensemble models exhibit similar performance profiles, with GradBoost maintaining competitive behavior across the full recall range. Importantly, PR curves show strong precision retention at moderate-to-high recall, indicating effective prioritization of active compounds during virtual screening.

**Figure 5:**
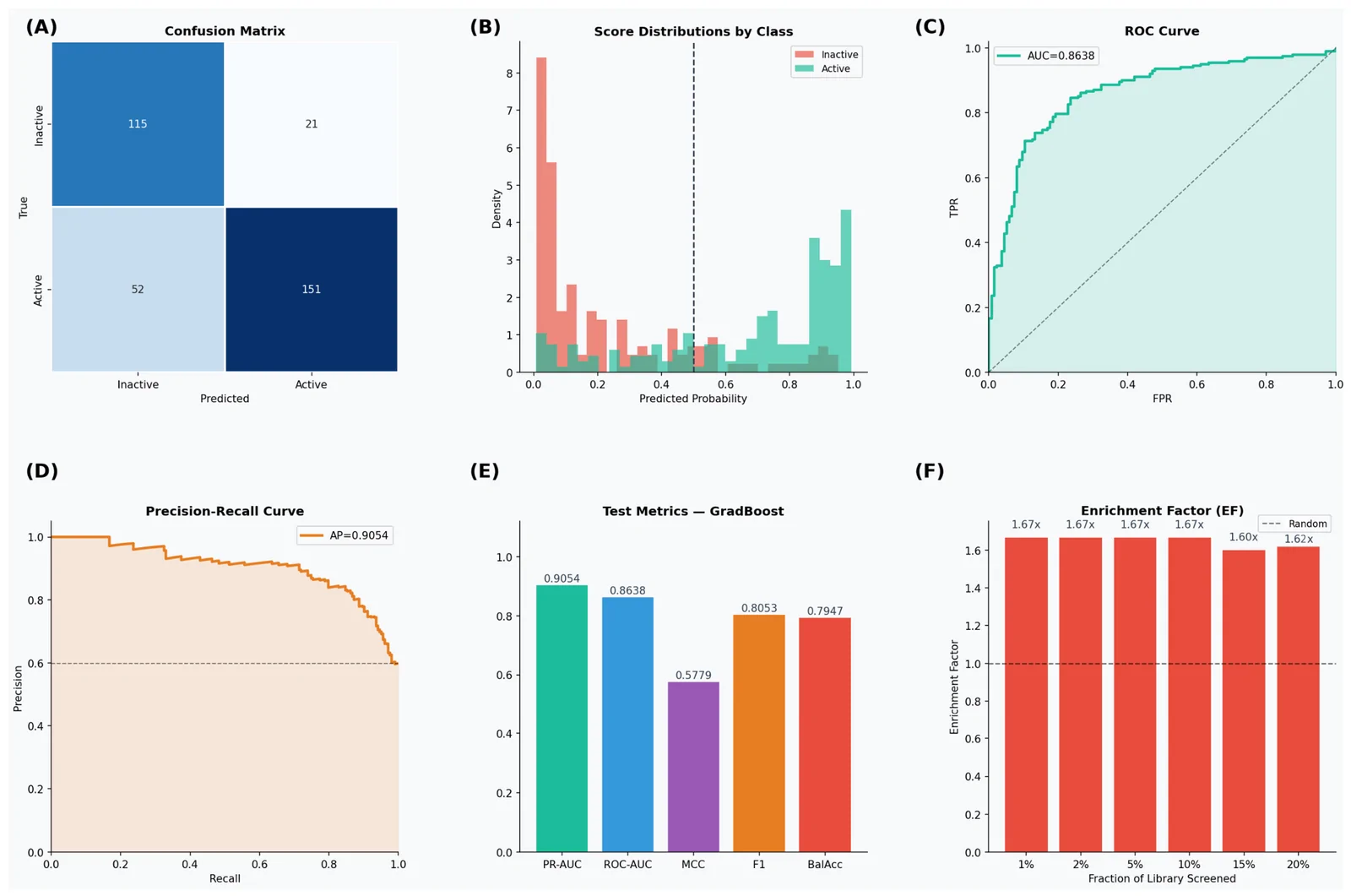
(**A**) Confusion matrix. (**B**) Predicted probability distributions by true class. (**C**) ROC curve with AUC. (**D**) PR curve with AP. (**E**) Test metric bar chart. (**F**) Enrichment factors.

A deeper inspection of GradBoost predictions reveals clear separation between active and inactive classes in probability space, although partial overlap remains, which explains the moderate MCC value. The confusion matrix shows a slightly higher number of false negatives than false positives, reflecting the inherent difficulty of identifying actives across unseen scaffolds. Enrichment factors were modest: EF (1%) = EF (5%) = EF (10%) = 1.67×, EF (15%) = 1.60×, EF (20%) = 1.62×. These values are constrained by the high baseline active fraction in the test set (59.9%); the theoretical maximum EF (1%) at this prevalence is only 1.67× (= 1/0.599), meaning the model achieves 100% enrichment efficiency in the top percentiles, with a near-perfect ranking of actives ahead of inactives. Overall, despite the challenges imposed by scaffold-disjoint evaluation, the proposed ERβ-Score demonstrates strong generalization, reliable ranking performance, and near-optimal early enrichment, supporting its applicability for prospective virtual screening of structurally novel compounds. The Confusion matrix predicted probability distributions by true class, ROC curve with AUC, PR curve with AP, Test metric bar chart, and Enrichment factors are shown in Figure 5A–F.

#### 2.4.3. Feature Importance and Cumulative Enrichment Analysis

An important analysis of the features using the GradBoost model revealed LogP (lipophilicity) as the dominant descriptor influencing ERβ activity. Other descriptors with significant influence include numerous PEOE_VSA descriptors, which provide information on partial charge distribution and molecular surface area. This finding implies the importance of hydrophobic interactions and electrostatic complementarity in determining ERβ activity. In addition, descriptors related to the 3D shape of molecules (NPR1 and PMI) and geometric indices (e.g., sphericity index) make significant contributions to the model, suggesting that molecular shape and spatial orientation are important factors in receptor binding. TPSA (topological polar surface area), MolWeight (molecular weight), and fractionCSP3 (fraction of carbon atoms attached to no more than one other atom) also add to the evidence that additional descriptors associated with polarity, size, and molecular flexibility further contribute to the model. Thus, we conclude that the ERβ-Score model can represent relationships in a structure–activity model not from a single property but from an interdependent collection of property measurements. Taken together, we conclude that the ERβ-Score model captures chemical similarities among active compounds and can therefore be very useful for virtual screening in practice, enabling rapid prioritization of large numbers of compounds before testing for activity. The results are shown in Supplementary Figure S4.

### 2.5. Y-Randomization Validation

**Figure 6:**
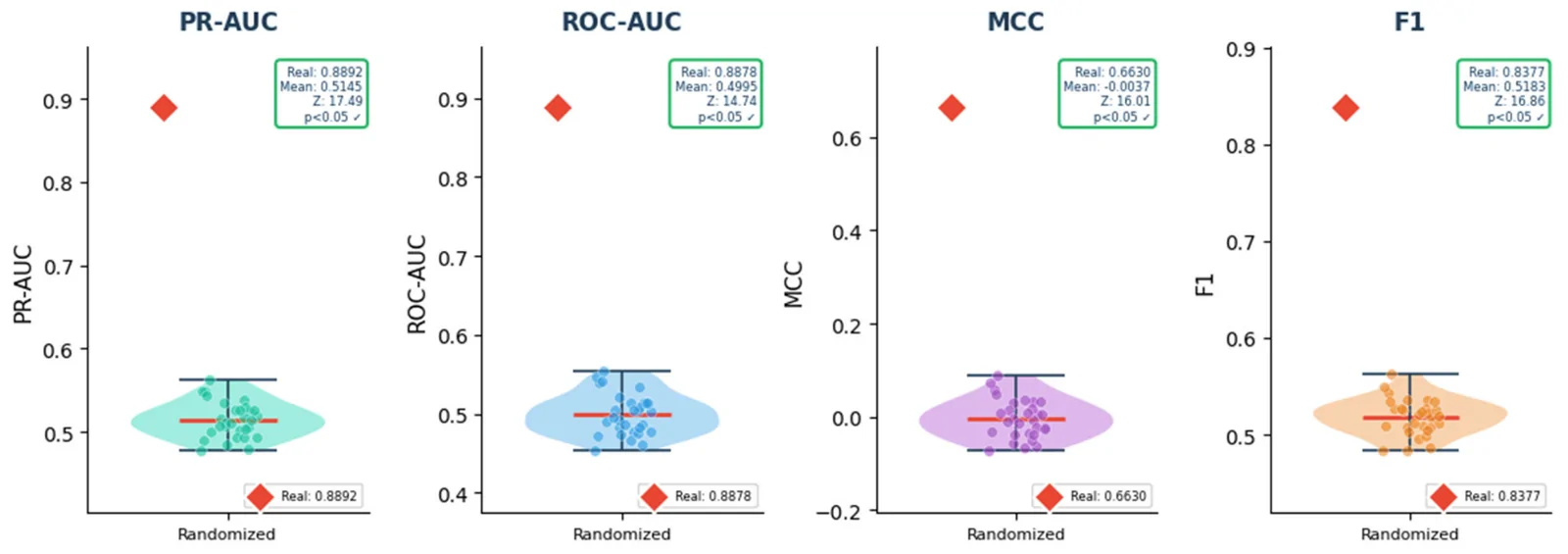
Y-randomization violin plots of randomized metric distributions (30 permutations) with real model values marked as red diamonds. Inset boxes show Z-scores and significance.

Y-randomization with 30 label permutations conclusively confirmed that ERβ-Score captures genuine structure–activity relationships. The real model OOF metrics were vastly separated from the randomized null distributions for all four metrics (Table 3). Z-scores ranged from 14.74 (ROC-AUC) to 17.49 (PR-AUC), all exceeding Z_crit_ = 1.645 by an order of magnitude (all *p* < 0.001). The randomized PR-AUC clustered around 0.5145 ± 0.0214 (near-chance for a 51.3%–active training set), while the real model achieved 0.8892, more than 17σ above the null. The randomized MCC distribution centered on −0.0037 ± 0.0416, confirming that permuted models show no predictive discrimination, whereas the real model’s MCC of 0.6630 represents strong concordance (Table 3). The results for the Y-randomization violin plots of randomized metric distributions (30 permutations) is given in Figure 6, while the Randomized distributions with fitted normal curves vs. real model values are shown in Supplementary Figure S5.

### 2.6. Applicability Domain Analysis

**Figure 7:**
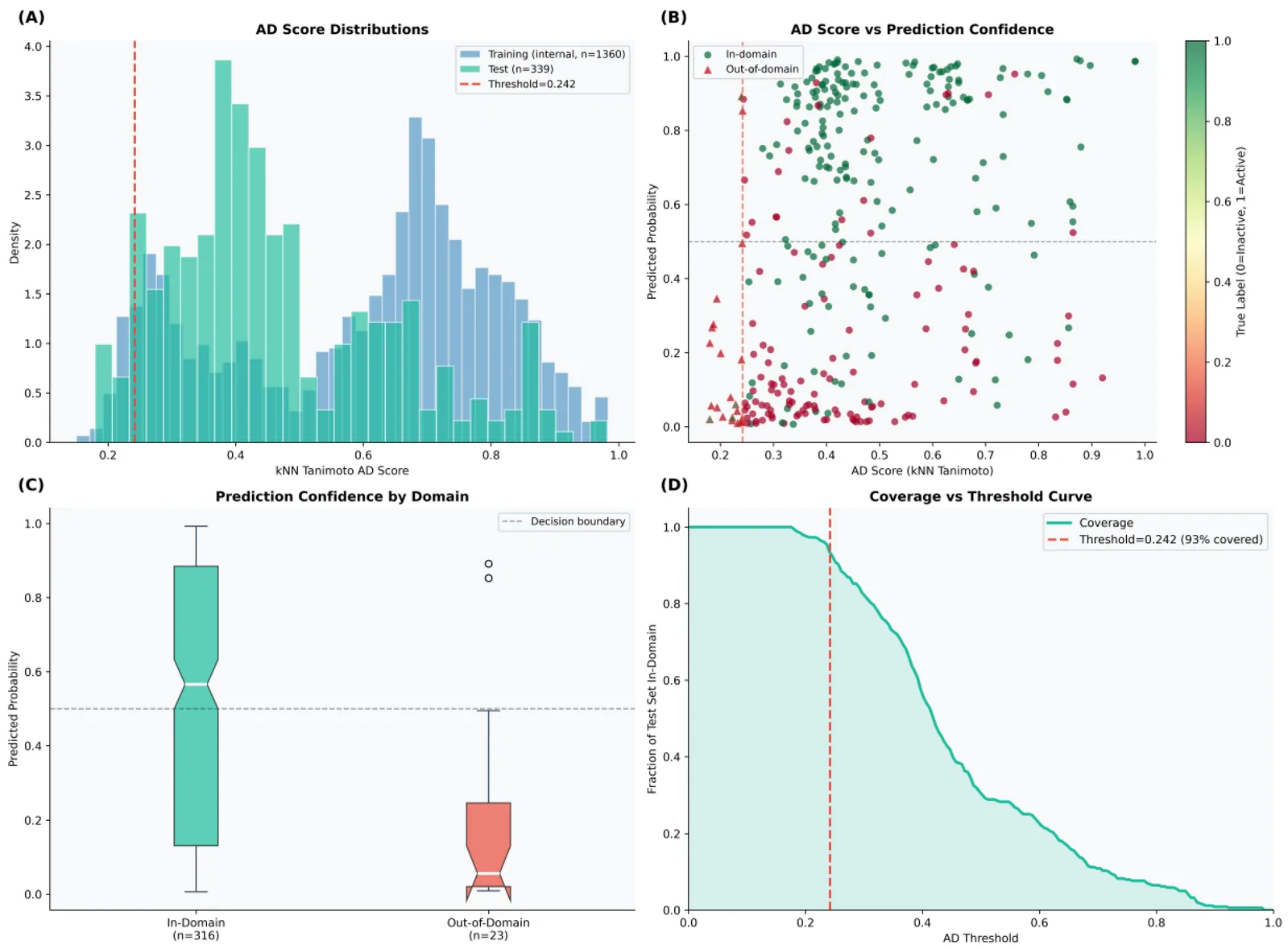
Applicability domain analysis. (**A**) AD score distributions for train/test. (**B**) AD score vs. predicted probability by true label. (**C**) Prediction confidence by domain. (**D**) Coverage–threshold curve.

**Figure 8:**
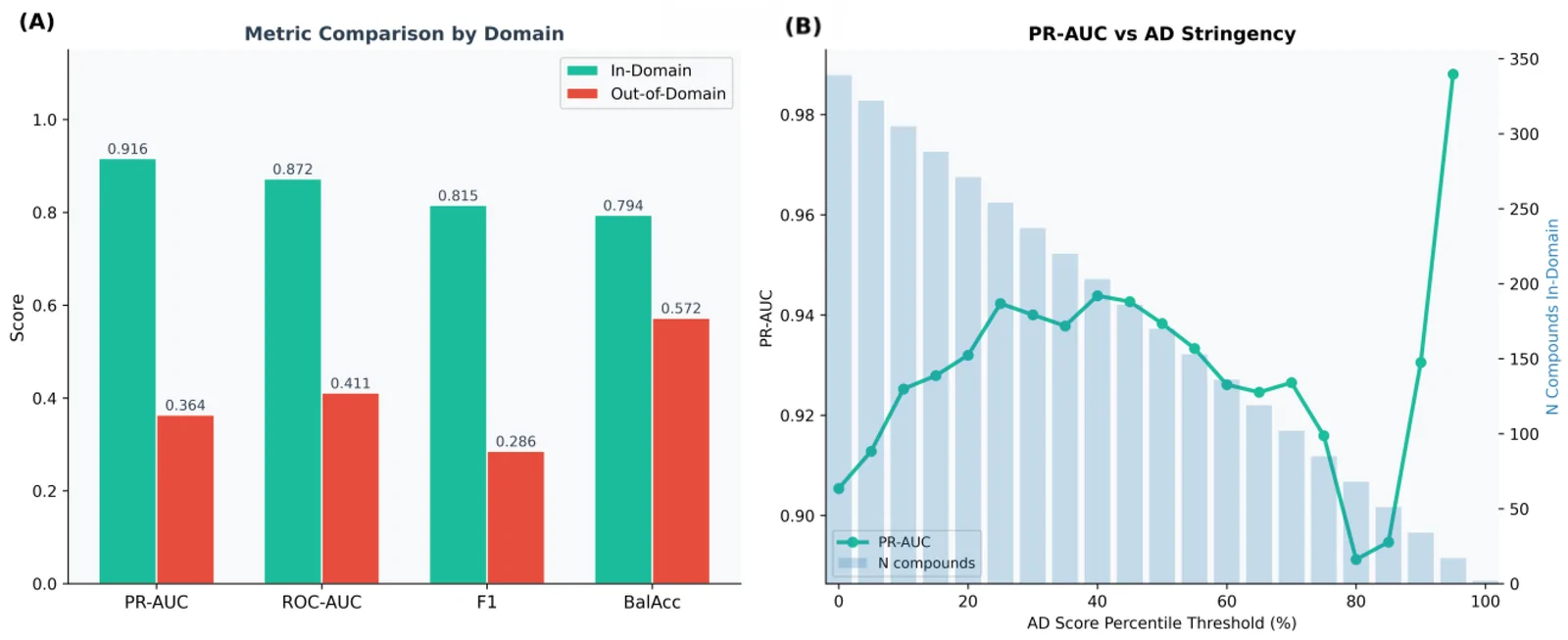
(**A**) Per-metric comparison between in-domain and out-of-domain. (**B**) PR-AUC vs. AD stringency with compound count overlay.

The kNN (*k* = 5) Tanimoto AD analysis yielded a threshold of 0.2419 (the 5th percentile of the training internal scores) (Table 4). The mean training AD score (0.6001 ± 0.2100) was substantially higher than the test-set mean (0.4613 ± 0.1752), consistent with the scaffold-disjoint splitting strategy that forces the test set into less densely populated regions of chemical space. Of 339 test compounds, 316 (93.2%) were classified as in-domain and 23 (6.8%) as out-of-domain. The high coverage indicates that, despite complete scaffold disjointness, the majority of test compounds reside within the training chemical-space envelope at the substructural level. Critically, model performance was strongly domain-dependent (Table 5). In-domain compounds achieved PR-AUC = 0.9161 and ROC-AUC = 0.8722, whereas out-of-domain compounds showed dramatically degraded performance (PR-AUC = 0.3638, ROC-AUC = 0.4111—near random). The F_1_-score dropped from 0.8152 (in-domain) to 0.2857 (out-of-domain), representing a 2.9-fold reduction. This sharp domain dependence underscores the necessity of AD assessment in prospective screening: predictions for out-of-domain compounds should be flagged and interpreted with caution. The AD framework thus serves as a built-in confidence estimator for ERβ-Score. The applicability domain analysis is shown in Figure 7A–D, while the Per-metric comparison between in-domain and out-of-domain and PR-AUC vs. AD stringency with compound count overlay is shown in Figure 8A,B. On the external Tox21 ERβ dataset, ESRβ-Score demonstrated statistically significant predictive performance above random classification (ROC-AUC = 0.709, 95% CI 0.686–0.732; Hanley–McNeil z = 16.0, *p* < 10^−55^), indicating that the model captures biologically relevant structure–activity relationships beyond the training data. However, predictive performance was reduced relative to the internal scaffold-holdout test (ROC-AUC = 0.864, MCC = 0.578), with an external MCC of 0.124 and sensitivity of 9.9% at the default classification threshold, whereas specificity remained high (97.6%). Bemis–Murcko scaffold analysis revealed that only 8.2% of external active compounds shared a scaffold with any training-set active, supporting the scaffold diversity imbalance observed in Figure 2B. Nevertheless, similarly low sensitivity for both scaffold-overlapping and scaffold-novel compounds suggests that differences in assay modality between the biochemical binding data used for model training and the live-cell functional reporter assay used for external validation also contributed to the reduced recall. Detailed compound-level results are provided in Supplementary Tables S2–S4.

### 2.7. Molecular Docking-Based Prioritisation

**Figure 9:**
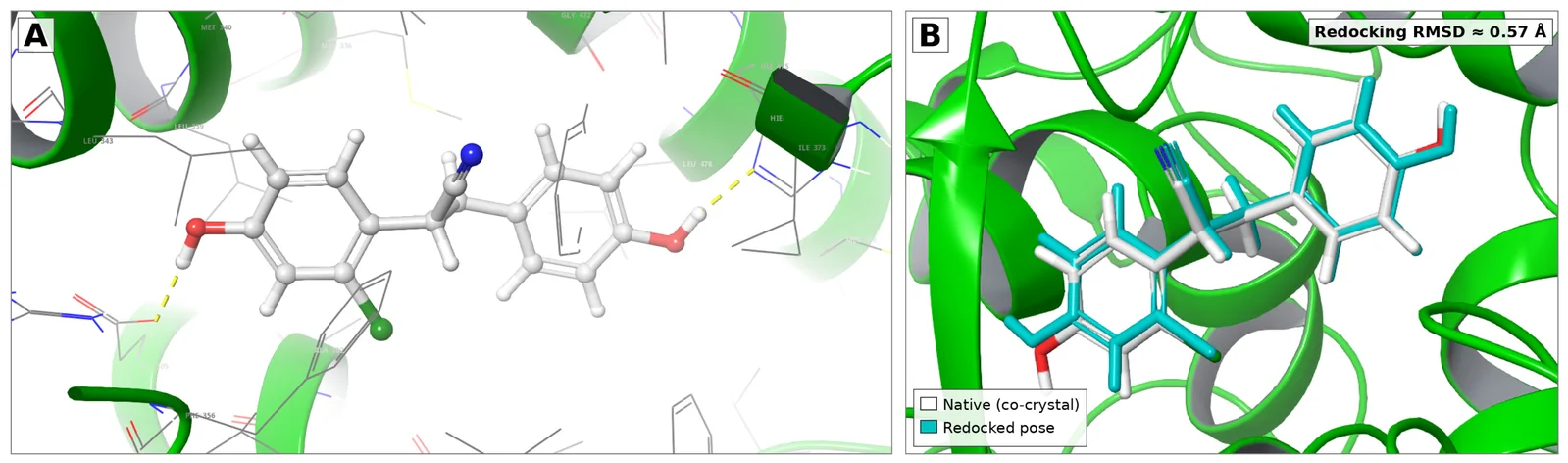
Docking-protocol validation against ERβ (PDB: 7XWQ). (**A**) Reference-ligand binding mode, with hydrogen bonds (yellow) to the conserved anchoring residues (incl. His475) and a hydrophobic enclosure. (**B**) Re-docked pose (cyan) superimposed on the native co-crystal ligand (white); heavy-atom RMSD ≈ 0.57 Å (<2.0 Å) confirms the protocol reproduces the experimental pose.

Structure-based prioritization was performed by docking the top virtual-screening hits from each library to ERβ (PDB: 7XWQ). The binding site was defined from the co-crystallized ligand coordinates (center: −3.24, −7.85, −39.60 Å; box: 22.5 × 22.5 × 22.5 Å). Docking was performed with Smina (exhaustiveness = 16, 9 modes, 8 CPUs); GNINA was not available on the compute node. The redocking validation results are shown in Figure 9A,B. The ERβ-Score model was applied to a large, chemically diverse virtual library comprising millions of compounds, which were screened using the ERβ-Score model from a diverse virtual database. Given the reduction in duplicate compounds, the ERβ-Score model identified 520,650 unique compounds, providing broad chemical coverage and minimal redundancy. The applicability domain (AD) was analyzed based on the number of unique compounds being classified in-domain (326,423 or 62.7%), confirming that the majority of predictions have high reliability. The assessments of chemical alignment from each library produced meaningful differences; for example, compounds within the ChEMBL library exhibited moderate predicted probabilities (mean approximately 0.23) for 2585 of the 2769 known actives that aligned with previously known bioactivity; IBScreen had the second lowest mean predicted probability (approximately 0.09) but produced a total of 12,110 hits an indication of the large size and diversity of the IBScreen library; NPAtlas had a mean predicted probability of approximately 0.14 and produced a total of 978 hits, indicating the presence of some novel natural-product-like chemotypes that have partial alignment to previously established ERβ SAR; and finally, through the greatly increased mean predicted probability of ERβ-relevant chemical features (mean in-domain approximately 0.67), ZINC compounds exhibited the highest level of hit discovery potential to ERβ. In conclusion, 15,950 active compounds were identified (approximately 3.1% of the total compound pool), suggesting that the ERβ-Score model can accurately and reliably estimate the biological activity of a library of potential compounds while serving as a calibration system for an effective predictive model. Smina docking was then applied as a second, structure-based prioritisation step, and the top-ranked candidate compounds showed favourable predicted binding scores (−10.94 to −8.85 kcal/mol). We emphasise that docking scores are computational estimates of pose complementarity and do not by themselves establish ERβ binding affinity, agonism, antagonism, or subtype selectivity in the absence of experimental measurement. Some of the highest-ranking compounds (ZINC26874, ZINC1565, ZINC5276, ERB-H1, ERB-H4, ERB-H5) provided both high predicted binding probabilities (>0.97) and good docking scores (−10 kcal/mol), while some compounds with intermediate or lower predicted probabilities still exhibited good to excellent docking energies. This illustrates the complementary nature of combining a machine learning approach with a docking-based approach for predicting binding, with machine learning providing a measure of overall global binding interactions and docking providing a measure of local binding interactions. The top-ranked compounds also remained within their respective applicability domains, which provides further support for their prioritization. Overall, these data indicate that the ERβ-Score pipeline integrates machine learning, applicability-domain filtering, and docking-based prioritisation to nominate a set of structurally diverse candidate ERβ ligands with favourable predicted binding scores for further experimental evaluation. With respect to hit selection, the compounds carried forward to docking were the top-ranked entries from each library after applicability-domain filtering, rather than a single probability cut-off applied across the pooled libraries; because the mean predicted probability differs markedly between libraries, this per-library selection necessarily retains some compounds with comparatively lower ML probabilities. Such compounds were deliberately retained to sample chemotypes structurally distinct from the training actives and to allow the orthogonal docking signal to be assessed independently of the ligand-based score, and they should be regarded as exploratory rather than prioritised candidates. Redocking of the 7XWQ co-crystallized ligand reproduced the native binding mode (heavy-atom RMSD = 0.570 Å), confirming that the docking set-up recovers the experimentally observed pose before its application to the prospective hits. The top hits are presented in Table 5.

### 2.8. ERβ-Score Web Application

**Figure 10:**
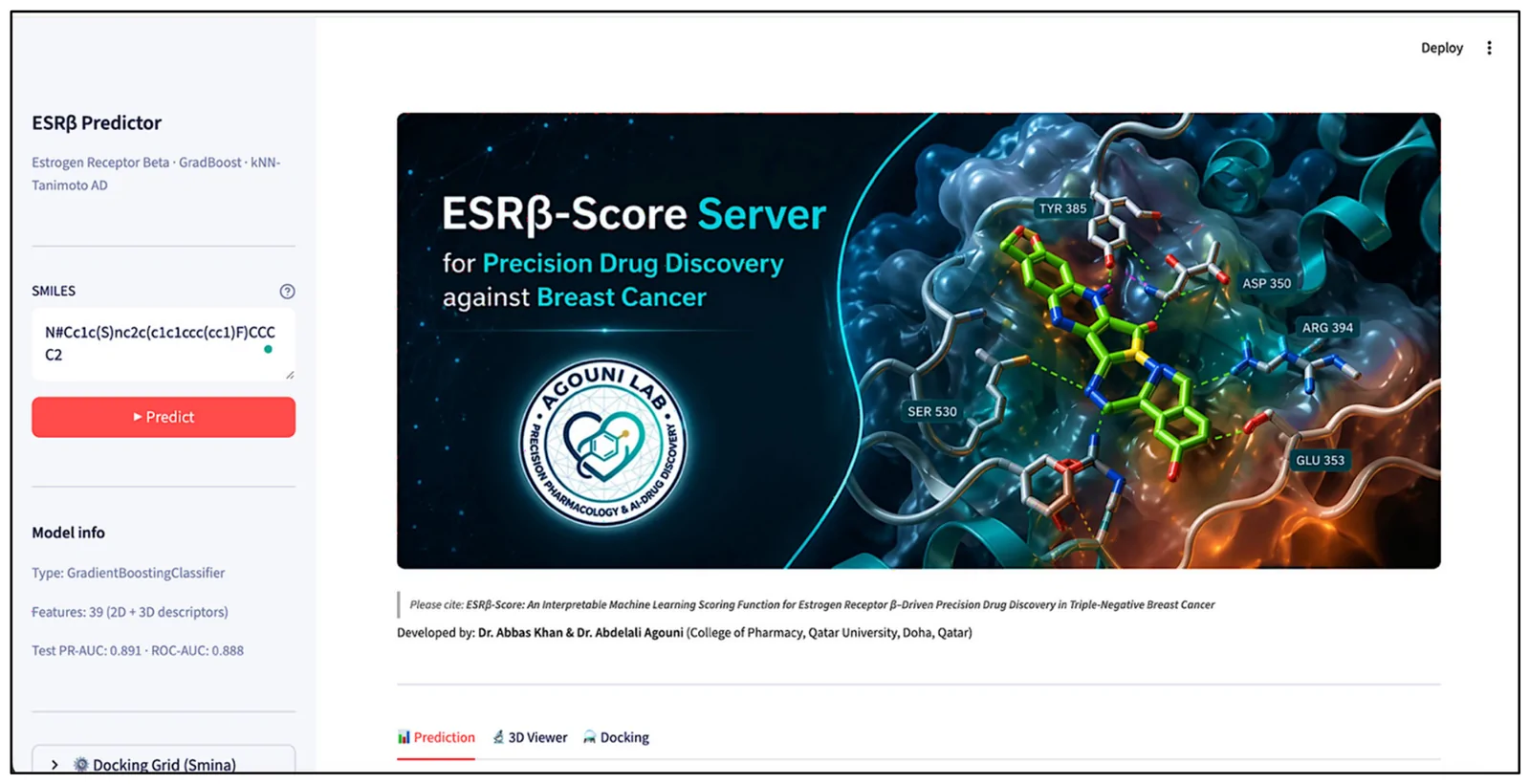
Frontend of the ERβ-score server, which can be used to predict the activity, ADMET properties, and perform docking with the ERβ receptor using smina.

We developed and deployed the ERβ-Score web application, providing a unified, single-page interface that integrates all stages of computational hit evaluation across three interactive tabs. Upon submission of a SMILES string, the Prediction tab returns an activity probability displayed as a semicircular gauge, a color-coded Active/Inactive classification badge, an applicability domain score visualized as a filled progress bar against the kNN-Tanimoto threshold (τ = 0.242), and a Lipinski Rule-of-Five summary table with pass/fail annotations for molecular weight, LogP, hydrogen bond donors and acceptors, topological polar surface area, and rotatable bond count. The 3D Viewer tab renders the query molecule’s MMFF94-optimized conformer, docked into the ERβ binding pocket, in real time, allowing interactive rotation, zoom, and translation directly within the browser without a plugin. The Docking tab executes Smina molecular docking with a single click, reports the best binding affinity in kcal/mol with an interpretive color scale (strong < −7, moderate −5 to −7, weak > −5 kcal/mol), and displays the docked complex in the same 3Dmol.js viewer alongside the full Smina scoring log. The docking grid parameters are pre-populated from the co-crystal structure but remain fully editable via a sidebar expander, supporting investigation of alternative binding sites, making the tool practical for rapid prioritization of virtual screening hits in ERβ-targeted drug discovery campaigns. Figure 10 shows the front end of the developed and deployed web application for bioactivity prediction against ERβ.

## 3. Discussion

These analyses present complementary evidence that defines both the predictive capability and the limitations of ERβ-Score. Using interpretable physicochemical and three-dimensional molecular descriptors, the ligand-based machine learning framework demonstrates that ERβ inhibitory activity, as represented by well-curated ChEMBL IC_50_ measurements, can be predicted accurately. The comparable performance among our tree-based algorithms, alongside Y-randomization, demonstrates that the predictive performance is rooted in meaningful structure–activity relationships rather than chance correlations. In addition, scaffold-disjoint validation indicates that model performance is maintained on compounds containing novel Bemis–Murcko scaffolds, supporting the conclusion that the classifier captures generalizable molecular features associated with ERβ activity rather than memorizing scaffold-specific patterns. Applicability-domain analysis helps to define the chemical space within which predictions can be considered reliable, while independent validation using a Tox21 ERβ functional bioassay provides an orthogonal assessment of the generalizability of the framework beyond the original training data. The accompanying web server further facilitates practical implementation of the complete prediction workflow.

Together, our results support the use of ERβ-Score as a computational prioritization framework for enriching compound libraries with candidate ERβ modulators, particularly for compounds located within the applicability domain. However, the findings do not provide evidence of biological efficacy or pharmacological activity. Independent validation confirmed statistically significant predictive performance (ROC-AUC = 0.709); however, the reduced sensitivity at the default classification threshold indicates lower recall for structurally diverse compounds evaluated using an independent functional assay. Additionally, the applicability-domain framework failed to consistently identify these prediction failures, indicating that chemical similarity alone is insufficient to ensure predictive reliability across different experimental platforms. A structure-based molecular docking approach provided complementary evidence by evaluating ligand compatibility within the ERβ ligand-binding pocket, and successful redocking replicated the crystallographic binding pose, supporting the validity of the docking protocol. However, docking scores provide computational estimates of binding affinity and should not be considered direct indicators of ligand binding or biological activity [23]. Consequently, the current computational analyses do not establish receptor selectivity, cellular potency, functional agonism or antagonism, or therapeutic efficacy in triple-negative breast cancer (TNBC), all of which require experimental confirmation through biochemical, cellular, and pharmacological assays.

Furthermore, the interpretability of ERβ-Score should be distinguished from prediction-level explainability. The chosen set of descriptors represents physicochemical variables, and global feature-importance analysis identifies molecular properties that contribute to overall model performance. However, global importance measures do not provide molecule-specific explanations for individual predictions and therefore cannot directly identify the structural determinants underlying a given prediction outcome. Because compound prioritization is performed at the individual molecule level, prediction-level interpretation would require complementary approaches such as SHAP, counterfactual explanations, or matched molecular pair analysis to quantify descriptor contributions for individual compounds. The inclusion of these approaches represents an important direction for the future development of the framework.

While interpreting the present findings, it is important to note several limitations. The training dataset comprises bioactivity measurements collected from multiple experimental sources and assay formats, which introduces unavoidable variation into the response labels. The results showed reduced predictive performance for compounds outside the applicability domain, while external validation further indicated reduced sensitivity for chemically novel active compounds, reflecting the combined influence of scaffold diversity and differences between biochemical binding assays and live-cell functional reporter assays. Therefore, the virtual screening and docking results should be considered hypothesis-generating rather than confirmatory. They provide a rationally prioritized set of chemically diverse compounds for experimental evaluation rather than validated ERβ modulators. Future work should focus on prospective biochemical binding assays, functional reporter assays, and cellular validation of the highest-ranked candidates, together with complementary ERα predictive models to enable quantitative assessment of receptor selectivity before translational application.

## 4. Materials and Methods

### 4.1. Dataset Compilation and Curation

We curated the bioactivity data for human ERβ from the ChEMBL database (https://www.ebi.ac.uk/chembl/ (accessed on 14 March 2026)) with the accession ID: CHEMBL242 [24]. For training purposes, entries reporting IC_50_ values (in nm) were used only for model training and downstream processing. All retained records corresponded to the human ERβ target (CHEMBL242) with standard_type = IC_h0_, standard_relation “=”, and standard_units of nM; bioactivities were aggregated across the available assay records and were not further stratified by assay format or cell system, a heterogeneity we acknowledge as a limitation and mitigated through the replicate-consistency filter and the wide activity gap defined below. Inactive compounds were therefore defined as experimentally weak ERβ binders (pIC_h0_ < 5.5) rather than untested or presumed-negative molecules, so that both classes derive from the same measured ERβ assay space and the classifier learns activity-discriminating rather than target-discriminating features. Before model training, the dataset was prepared, and any entry with records containing ChEMBL ID, canonical SMILES, and, particularly, the standard value, which represents the IC_50_ values, was excluded. Moreover, the Lipinski molecular-weight-based criteria were applied to remove compounds with higher molecular weights. An algorithmic conversion of IC_50_ to pIC_50_ (=9 − log_10_(IC_50_ [nM])) was performed to standardize the protocol for machine learning processing. Exact-duplicate rows and records sharing identical (ChEMBL ID, IC_50_) pairs were deduplicated. Compounds with multiple measurements exhibiting pIC_50_ standard deviation > 0.5 log units were excluded as inconsistent; for concordant replicates, the entry nearest the median pIC_50_ was retained. A binary classification framework was implemented with a deliberate intermediate-exclusion gap to suppress label noise. Compounds with pIC_50_ ≥ 7.5 (IC_50_ ≤ 31.6 nM) were assigned Active = 1; those with pIC_50_ < 5.5 (IC_50_ > 3.16 μM) were assigned Inactive = 0. Compounds in the intermediate zone (5.5 ≤ pIC_50_ < 7.5) were excluded to reduce class-boundary ambiguity. The purpose was to include the strong actives and strong inactives in the training, so the prediction of new compounds is strictly activity-associated. The pIC_h0_ ≥ 7.5 and <5.5 cut-offs were set to exceed the typical inter-laboratory uncertainty of IC_h0_ measurements (commonly ~0.5–1 log unit), so that the assigned active and inactive labels are robust to experimental noise; the intervening 2-log buffer is a deliberate trade-off that prioritizes label fidelity over coverage of borderline compounds.

### 4.2. Molecular Representation

To produce a set of molecular fingerprints using various methods that capture a diversity of structural features, two types of extended connectivity fingerprints (ECFP4 and ECFP6) were calculated using the Morgan algorithm (radius = 2 and 3, respectively), resulting in 2048 bits each [25,26]. The MACCS keys (166 bits) were computed according to the standard MACCS fingerprinting method. Furthermore, the RDKit topological fingerprints were calculated with a maximum path length of 7 and a size of 2048 bits [27,28]. Additionally, a panel consisting of 44 two-dimensional descriptors calculated from RDKit was created, which included six families based on the types of descriptors used, such as physicochemical descriptors (MolWt, LogP, TPSA, NumHBD, NumHBA, NumRotatableBonds, NumAromaticRings, RingCount, FractionCSP3, HeavyAtomCount), Kier-Hall connectivity indices (Chi0n-Chi4n), Kier shape indices (Kappa1-Kappa3), topological indexes (BalabanJ), EState VSA indexes (EState_VSA1-EState_VSA11), and PEOE VSA indexes (PEOE_VSA1-PEOE_VSA14). Three-dimensional conformations were generated for the molecules using the ETKDG v3 stochastic distance geometry algorithm (AllChem.EmbedMolecule with ETKDGv3 parameters) followed by force-field optimization using the MMFF94 force field, with NaN assigned to any molecules that failed embedding (return code = 1). A total of 10 three-dimensional descriptors were calculated, including principal moments of inertia (3 total PMI1, PMI2, PMI3), normalized PMI ratios (2 total NPR1, NPR2), radius of gyration, asphericity, eccentricity, inertial shape factor (2 total), and sphericity index [29]. Therefore, the resulting descriptor matrix combined the two- and three-dimensional systems for each compound, yielding 54 total descriptive features per compound (44 two-dimensional and 10 three-dimensional). For all these calculations, RDKit [27] was used.

### 4.3. Feature Preprocessing Pipeline

A 54-feature descriptor matrix generated by RDKit was processed sequentially in a four-step pipeline using scikit-learn 1.3+ with a random seed of 42 [30]. Missing values were imputed using the median, followed by the removal of zero-variance features and near-zero-variance features (variance < 0.01). Finally, highly collinear features were eliminated by removing one variable from each pair with Pearson correlation |r| > 0.95. Descriptors were thus retained on objective, unsupervised criteria—interpretability, low missingness, and non-redundancy (near-zero-variance and pairwise-correlation filters) rather than by activity-informed selection, which avoids feature-selection bias and label leakage into the descriptor set.

### 4.4. Scaffold-Disjoint Data Splitting

The Bemis–Murcko scaffolds were extracted from the given dataset using RDKit with MurckoScaffoldSmiles, including Chirality = False, to represent core chemical frameworks [31]. To rigorously evaluate model generalization across distinct chemical classes, a scaffold-disjoint splitting strategy was employed. Bemis–Murcko scaffold groups were randomly permuted and assigned to the test set until approximately 20% of the compounds were included, ensuring that no scaffold was shared between the training and test partitions. Within the training set, we used a stratified five-fold cross-validation approach with StratifiedKFold (n_splits = 5, shuffle = True) to ensure class balance during model development.

### 4.5. Algorithm Ensemble and Bayesian Hyperparameter Optimization

A comprehensive model development strategy was implemented using ten state-of-the-art machine learning algorithms, including Random Forest (RF), Extra Trees (ET), XGBoost, LightGBM, Histogram-based Gradient Boosting (HistGradBoost), Gradient Boosting (GradBoost), support vector machines with radial basis function kernel (SVM-RBF), Multi-Layer Perceptron (MLP), Logistic Regression (LR), and k-Nearest Neighbors (kNN), to ensure broad coverage of both ensemble-based and classical learning paradigms [32]. The stochastic models were initialized with the same fixed random seed (42) for consistent results across runs, and the tree model calculations used multiple processors (n_jobs = −1) when available to maximize processing time. All hyperparameter optimizations used Optuna with Tree-structured Parzen Estimator (TPE) Bayesian optimization (BayesOpt) on each of the algorithms in 20 optimization trials per algorithm with a fixed TPE seed (42) and with the objective defined as maximizing the mean out-of-fold PR-AUC across the 5 stratified cross-validation folds, which is important due to the imbalanced nature of the classification task. To avoid data leakage, all data standardization performed by StandardScaler was applied independently to each training fold across all optimization trials. The search spaces for hyperparameter optimization were defined to balance exploration and computation time, optimizing the number of iterations required for training each algorithm. Some of the parameters that were optimized in the Random Forest (RF) and Extra Trees (ET) models included the number of estimators (100–600), maximum depth of tree (3–20), the minimum number of samples required before a split (and for leaf nodes), and the tree feature subsampling methods (‘sqrt’, ‘log2’, or fractions). For XGBoost, the hyperparameters were tuned for the number of estimators, maximum depth, learning rate (log-uniform), subsampling ratios, and regularization parameters (gamma, L1, and L2 penalties). LightGBM optimization similarly covered tree complexity (num_leaves, depth), learning rate, subsampling, and regularization terms. Gradient boosting variants (HistGradBoost and GradBoost) were tuned across iterations, depth, learning rates, subsampling, and regularization. For SVM-RBF, the regularization parameter (C) and kernel width (γ) were optimized over logarithmic ranges, while MLP architectures were explored by varying network depth (1–3 layers), hidden units (32–512), learning rate initialization, and L2 regularization strength. Logistic regression models were tuned over regularization strength (C) and penalty type (L1 or L2), and kNN models were optimized for neighborhood size, weighting scheme, and distance metric. This systematic, harmonized optimization framework ensured a fair and rigorous comparison across diverse algorithms while maximizing predictive performance under controlled, reproducible conditions. The detailed parameter settings for each algorithm, including hyperparameters and search ranges, are provided in Supplementary Table S1.

### 4.6. Model Selection and Evaluation

We analyzed models’ performance using five complementary metrics and evaluated them across each cross-validation fold to quantify both ranking and classification quality [33]. The primary evaluation criterion for this study is the precision–recall area under the curve (PR-AUC), as calculated by the average precision formulation. Average precision offers the most reliable estimate of performance on an imbalanced dataset, as it emphasizes correct identification of the positive (active) class [34]. Global ranking performance was assessed using the receiver operating characteristic area under the curve (ROC-AUC) statistic. Finally, the Matthews correlation coefficient (MCC), F1-score, and balanced accuracy were all calculated as classification metrics based on predictions using the 0.5 fixed decision threshold. The MCC, which accounts for all elements of the confusion matrix, was calculated as:

(1) $$MCC=\frac{TP\times TN-FP\times FN}{\surd (TP+FP)(TP+FN)(TN+FP)(TN+FN)}$$

In the above equation, TP, TN, FP, and FN represent true positives, true negatives, false positives, and false negatives, respectively. The F1-score, representing the harmonic mean of precision and recall, was computed as:

(2) $$F1=\frac{2\times TP}{2\times TP+FP+FN}$$

Balanced accuracy was defined to account for class imbalance by averaging sensitivity and specificity:

(3) $$Balanced\;Accuracy=\frac{1}{2}\left(\frac{TP}{TP+FN}+\frac{TN}{TN+FP}\right)$$

Following the cross-validation, a model with the maximum mean PR-AUC across folds was selected as optimal. This model was retrained on the full training data using a single StandardScaler applied to all training samples. It was then assessed against the Scaffold-disjoint independent test set. The early recognition performance of a single model, calculated at different cut-off values (1%, 2%, 5%, 10%, 15%, and 20%), was assessed for virtual screening scenarios. The EF for a given fraction is defined as:

(4) $${EF}_{x\%}=\frac{{Hits}_{top\;x\%}}{\frac{N_{topx\%}}{\frac{N_{active}}{N_{total}}}}$$

Hits_top_ x% is the number of active compounds found within the top x%, N_top_ x% is the total number of compounds within the top x%, N_active_ is the number of active compounds in the dataset, and N_total_ is the total number of compounds in the dataset. This measure assesses how well the model enriches for active compounds at the top of the ranked list relative to a random selection of compounds.

### 4.7. Y-Randomization Validation

The robustness of the predictive models was assessed to exclude the possibility of chance correlations. For this purpose, Y-randomization (response permutation testing) was performed on the training labels [35,36]. The labels were randomly permuted *N_rand_* = 30 times using NumPy’s v2.5.0 RandomState. For each permutation, the selected best-performing model (based on cross-validation) was retrained under identical conditions, including the same hyperparameters, cross-validation folds, preprocessing pipeline, and random initialization. Model performance was evaluated using out-of-fold predictions obtained from five stratified cross-validation folds. The same performance metrics used in the original evaluation, PR-AUC, ROC-AUC, Matthew’s correlation coefficient (MCC), and F1-score, were computed for each randomized iteration.

From each matrix, we calculated the Z score to determine the statistical significance of the observed performance as

(5) $$Z=\frac{m_{real}-\mu_{rand}}{\sigma_{rand}}$$

where m_real_ denotes the performance metric resulting from the original (non-permuted) model and µ_rand_ and σ_rand_ represent the mean and standard deviation of the corresponding metric over the randomized runs. One-sided *p*-values were calculated using the survival function of the standard normal distribution:

(6) ρ=1−ϕ(Z)

The cumulative distribution function (CDF) of the normal distribution is represented by ϕ(Z). The level of statistical significance used to evaluate the results of this analysis was predetermined at 0.05. The significant difference between the model’s actual performance and the distribution resulting from randomization, as reflected in Z-scores and *p*-values, provides evidence that the model successfully estimates the true structure of activity relationships rather than spurious correlations.

### 4.8. Applicability Domain Analysis and External Validation

The AD was defined using *k*-nearest-neighbor (*k* = 5) Tanimoto similarity on ECFP4 fingerprints (2048 bits) [37,38]. For each query compound *q*, the following equation was used to calculate the AD score.

(7) $$AD(q)=\frac{1}{k}\sum_{i=1}^{k}T_{c}(q,\;nni)\;$$

Using an analogous leave-one-out process on the training data to determine the internal AD score distribution, a threshold was set at the 5th percentile of the training AD scores. Test compounds with an AD score ≥ threshold were classified as being in-domain, and all other test compounds were classified as being out-of-domain. Model performance was independently measured in each domain. To evaluate generalizability beyond the curated ChEMBL training distribution, we performed an external validation using two independent Tox21 estrogen receptor beta (ERβ) reporter-gene qHTS bioassays obtained from the NCATS open-data portal (agonist mode, PubChem AID 1259394; antagonist mode, AID 1259378), neither of which was part of our ChEMBL-derived curation pipeline. Per-replicate results were consolidated to a consensus call per compound; compounds confirmed active in either assay were labeled Active, compounds inactive in both were labeled Inactive, and compounds with inconclusive or disagreeing replicate calls were excluded rather than force-labeled. After structure canonicalization and removal of 33 compounds overlapping our training/test set, the final external set comprised 5913 compounds (525 Active, 5388 Inactive). Predictions were generated using the saved production model, scaler, and applicability-domain gate with no retraining.

### 4.9. Prospective Virtual Screening and Validation

The best-performing classifier, along with the fitted StandardScaler, selected features, median imputation parameters, and the applicability domain (AD) configuration, was serialized using joblib and JSON. The four external libraries evaluated were TCM, NPAtlas, IBScreen, and ChEMBL-derived and ZINC [39,40,41,42]. All four libraries have undergone the same SMILES parsing, feature alignment via the 2D/3D descriptor pipeline, feature imputation using median values from training datasets, and computation of their respective class probability values (P_active_) and AD scores. All processes were conducted by deduplicating the output based on canonical SMILES while retaining the highest-class probability (P_active_). The top 10 hits for docking against ERβ were selected from the top 10 compounds in each library (prioritized within their respective domains). The ligand’s 3D structure was generated using ETKDG v3 + MMFF94 in RDKit and converted into PDBQT using Open Babel [43]. The receptor was prepared by extracting ATOM/TER records from the PDB and stripping the HETATM records [44]. The docking box was centered on the co-crystallized ligand’s centroid, with a minimum of 8 Å padding per axis (minimum of 22.5 Å). Smina (an AutoDock Vina fork) was used to perform the docking of the selected ligands with the ERβ with an exhaustiveness level of 16, a binding mode of 9, and 8 CPU threads [45]. To validate the docking protocol, the co-crystallized ligand of 7XWQ was re-docked into the prepared binding site under identical Smina settings, and pose reproducibility was quantified as the heavy-atom RMSD between the top-scored pose and the crystallographic conformation (redocking RMSD = 0.570 Å); an RMSD ≤ 2.0 Å is conventionally regarded as a validated setup.

### 4.10. ERβ-Score Web Application Development

The ERβ-Score prediction pipeline was deployed as an interactive web application using the Streamlit framework (v1.35+), enabling real-time inference without requiring users to install or configure any local cheminformatics environment. The application loads a pre-trained Gradient Boosting Classifier, along with its associated StandardScaler, feature column definitions, training-set median values, applicability domain configuration, and ECFP4 fingerprint matrix from serialized artifacts, all cached server-side using Streamlit’s cache_resource mechanism to avoid redundant I/O on each user interaction. Molecular input is accepted as a SMILES string, from which 39 physicochemical descriptors are computed on-the-fly via RDKit, including 3D shape descriptors (PMI1, PMI2, NPR1, Spherocity Index) derived from ETKDGv3/MMFF94-optimized conformers, with automatic median imputation applied to any descriptor that fails due to embedding or computation errors. Applicability domain assessment is performed using a vectorized NumPy implementation of kNN-Tanimoto similarity against the 1360-compound training fingerprint matrix. Protein–ligand three-dimensional visualization is rendered directly in the browser via an embedded 3Dmol.js HTML component, displaying the ERβ receptor (PDB: 7XWQ) as a cartoon with the query ligand superimposed as element-colored sticks. Structure-based docking is executed on demand through a subprocess call to Smina, using a pre-computed binding grid centered on the co-crystallized ligand coordinates (center: −3.244, −7.848, −39.600 Å; box: 22.5 × 22.5 × 22.5 Å), with the docked complex subsequently rendered in the same interactive 3D viewer. The application is fully self-contained and can be launched locally or deployed to any cloud platform supporting Python environments via streamlit run app.py.

### 4.11. Software and Reproducibility

All analyses were implemented in Python 3.10+ (in a conda environment). Key dependencies: RDKit 2023.09+ (cheminformatics), scikit-learn 1.3+ (ML/preprocessing), XGBoost 1.7+, LightGBM 4.0+, Optuna 3.0+ (HPO), Smina/GNINA v1.2 (docking), Open Babel 3.1+ (format conversion), Matplotlib 3.7+/Seaborn 0.12+ (visualization). A global random seed of 42 was enforced across all stochastic operations.

## 5. Conclusions

In this study, we developed and validated the ERβ-Score, a machine learning-based scoring system aimed at expediting the identification of ERβ modulators for TNBC drug discovery. Through the integration of scaffold-disjoint data partitioning, meticulous feature engineering utilizing two-dimensional physicochemical and three-dimensional molecular shape descriptors, alongside Bayesian hyperparameter optimization, the Gradient Boosting Classifier demonstrated consistently robust predictive performance across all evaluation metrics. This validates that significant structure–activity relationships influencing ERβ binding can be derived solely from physicochemical descriptors, eliminating the need for computationally intensive quantum-mechanical or deep learning-based models. The integration of a kNN-Tanimoto applicability domain framework mitigates a significant limitation of prevalent bioactivity prediction models by providing users with a clear, quantitative delineation of prediction reliability, thereby ensuring that high-confidence predictions are differentiated from extrapolations into unexplored chemical space. External validation against independent Tox21 ERβ bioassay data confirmed that ESRβ-Score retains statistically significant discriminative power outside its training distribution but also revealed a meaningful reduction in sensitivity for structurally novel and assay-divergent active compounds. This finding substantiates a limitation inherent to the scaffold-imbalanced training data and underscores that ESRβ-Score is best used as a ranking and triage tool within its documented applicability domain, rather than a stand-alone binary classifier, for prospective screening decisions. The virtual screening of structurally diverse compound libraries using ERβ-Score identified a selection of high-probability candidates, which were later validated through structure-based docking against the ERβ co-crystal structure, demonstrating the scoring function’s efficacy as an initial filter in multi-stage computational workflows. The launch of a comprehensive prediction, applicability domain, drug-likeness, and docking workflow as an openly accessible Streamlit web application marks a significant advancement in democratizing computational drug discovery tools, eliminating the technical obstacles that often hinder experimental pharmacologists and medicinal chemists from using machine learning models in their research.

In summary, the ERβ-Score provides a consistent and interpretable computational framework that integrates ligand-based and structure-based methodologies for compound prioritisation. Its outputs are predictions of ERβ potency and should be treated as a starting point for experimental investigation rather than as evidence of functional activity, receptor selectivity, or therapeutic relevance in TNBC. We expect it to be an important resource for the wider community engaged in developing novel ERβ-selective therapeutics for TNBC and other estrogen-driven malignancies. Future endeavors will concentrate on augmenting the training dataset with newly available ChEMBL bioactivity data, integrating explainability analyses, such as SHAP feature attribution, to elucidate the structural determinants of ERβ selectivity, and on prospective experimental validation of the highest-ranked virtual screening candidates through in vitro binding experiments and cellular assays. We further note three boundaries that define the current scope of ERβ-Score: first, all predictions are computational and await prospective experimental confirmation, which is ongoing; second, the model scores ERβ potency rather than ERβ-over-ERα selectivity, so selective-modulator prioritization will require dedicated counter-target (ERα) models, which we regard as the most valuable next step; and third, predictive reliability is formally bounded by the applicability domain, with markedly reduced performance for out-of-domain chemotypes, underscoring that the tool is best used as an enrichment filter within rather than a replacement for integrated experimental pipelines.

## Figures and Tables

**Table 1:** Five-fold cross-validation results (mean ± SD). Best values per metric are highlighted in green. GradBoost selected as ERβ-Score by PR-AUC.

Model	PR-AUC	ROC-AUC	MCC	F1	BalAcc
**GradBoost**	0.8911 ± 0.021	0.8882 ± 0.011	0.6637 ± 0.032	0.8378 ± 0.014	0.8312 ± 0.017
**XGBoost**	0.8905 ± 0.023	0.8883 ± 0.009	0.6666 ± 0.020	0.8390 ± 0.007	0.8327 ± 0.011
**RandomForest**	0.8898 ± 0.028	0.8885 ± 0.014	0.6466 ± 0.029	0.8326 ± 0.013	0.8219 ± 0.015
**ExtraTrees**	0.8891 ± 0.025	0.8889 ± 0.014	0.6619 ± 0.050	0.8408 ± 0.022	0.8291 ± 0.026
**LightGBM**	0.8874 ± 0.021	0.8872 ± 0.007	0.6564 ± 0.026	0.8348 ± 0.011	0.8274 ± 0.012
**HistGradBoost**	0.8873 ± 0.019	0.8855 ± 0.010	0.6503 ± 0.027	0.8330 ± 0.013	0.8244 ± 0.014
**SVM-RBF**	0.8863 ± 0.022	0.8885 ± 0.012	0.6679 ± 0.028	0.8398 ± 0.013	0.8335 ± 0.014
**MLP**	0.8618 ± 0.030	0.8695 ± 0.018	0.6097 ± 0.035	0.8067 ± 0.020	0.8046 ± 0.021
**kNN**	0.8514 ± 0.025	0.8617 ± 0.015	0.5399 ± 0.041	0.7949 ± 0.019	0.7565 ± 0.024
**LogisticReg**	0.8195 ± 0.028	0.8349 ± 0.020	0.5369 ± 0.038	0.7890 ± 0.021	0.7631 ± 0.019

**Table 2:** ERβ-Score (GradBoost) test-set performance on the scaffold-disjoint hold-out (n = 339).

Model	PR-AUC	ROC-AUC	MCC	F1-Score	Balanced Accuracy
RandomForest	0.9067	0.8687	0.6106	0.8338	0.8096
ExtraTrees	0.9109	0.8754	0.6037	0.8316	0.8059
XGBoost	0.8985	0.8576	0.5541	0.7836	0.7824
LightGBM	0.8987	0.8562	0.5272	0.7796	0.7689
HistGradBoost	0.8887	0.8529	0.5414	0.8010	0.7752
**GradBoost**	**0.9054**	**0.8638**	**0.5779**	**0.8053**	**0.7947**
SVM_RBF	0.9173	0.8650	0.6017	0.7955	0.8056
MLP	0.8722	0.8530	0.5708	0.8144	0.7899
LogisticReg	0.7703	0.7701	0.4664	0.7933	0.7299
KNN	0.8902	0.8327	0.4405	0.7991	0.7043

**Table 3:** Y-randomization statistical summary (N = 30 permutations). All metrics are significantly different from the randomized null (*p* < 0.001).

Metric	Real Model	Rand. Mean ± Std	Z-Score	*p*-Value	Sig.
**PR-AUC**	0.8892	0.5145 ± 0.0214	17.49	<0.001	YES
**ROC-AUC**	0.8878	0.4995 ± 0.0263	14.74	<0.001	YES
**MCC**	0.6630	–0.0037 ± 0.0416	16.01	<0.001	YES
**F1**	0.8377	0.5183 ± 0.0189	16.86	<0.001	YES

**Table 4:** Per-domain model performance. In-domain compounds show markedly superior prediction reliability.

Domain	N	Active %	PR-AUC	ROC-AUC	F1	BalAcc
**All Test**	339	59.9%	0.9054	0.8638	0.8053	0.7947
**In-Domain**	316	62.7%	0.9161	0.8722	0.8152	0.7940
**Out-of-Domain**	23	21.7%	0.3638	0.4111	0.2857	0.5722

**Table 5:** Results of top hits from different databases reported as active by the ERβ-Score scoring function validated by Smina.

Rank	ID	ML Probability	AD Score	Domain	Smina (kcal/mol)
1	ZINC26874	0.927 (Active)	In-Domain	−10.691	1
2	ZINC1565	0.927 (Active)	In-Domain	−10.687	2
3	ZINC5276	0.925 (Active)	In-Domain	−10.664	3
4	ERB-H1	0.975	In-domain	−10.94	4
5	ERB-H2	0.809	In-domain	−10.61	5
6	ERB-H3	0.443	In-domain	−10.18	6
7	ERB-H4	0.984	In-domain	−10.12	7
8	ZINC6820	0.957 (Active)	In-Domain	−9.675	8
9	ERB-H5	0.983	In-domain	−9.58	9
10	ZINC4726	0.891 (Active)	In-Domain	−9.583	10
11	ZINC753	0.891 (Active)	In-Domain	−9.579	11
12	ZINC25978	0.891 (Active)	In-Domain	−9.538	12
13	ERB-H6	0.442	In-domain	−9.52	13
14	ERB-H7	0.662	In-domain	−9.41	14
15	ZINC25945	0.893 (Active)	In-Domain	−9.391	15
16	ERB-H8	0.465	In-domain	−9.10	16
17	ERB-H9	0.011	In-domain	−8.89	17
18	ERB-H10	0.067	In-domain	−8.85	18
19	ZINC5384	0.897 (Active)	In-Domain	−8.739	19
20	ZINC12219	0.895 (Active)	In-Domain	−8.658	20

## Data Availability

The complete curated dataset—including ChEMBL identifiers, canonical SMILES, measured IC_h0_/pIC_h0_ values, and binary activity labels for all 1699 modeling compounds—together with the full descriptor matrix, the serialized model artifacts, and the analysis and web-application source code, has been deposited in a public repository (GitHub: https://github.com/Abbas24-AI/ESR-beta-scoring-function-AI (accessed on 14 March 2026)). The ERβ co-crystal structure is available from the RCSB Protein Data Bank under accession code 7XWQ. All authors confirm that all data in this manuscript are an accurate and transparent account of the study being reported. The server is available at: https://esr-beta-scoring-function-ai.streamlit.app/ (accessed on 14 March 2026).

## Supplementary Materials

The following supporting information can be downloaded at: https://www.mdpi.com/article/10.3390/ijms27167089/s1.
