# Supplementary material for: ERβ-Score: An Interpretable Machine Learning-Based Scoring Function and Web Server for Estrogen Receptor β-Guided Drug Discovery in Triple-Negative Breast Cancer
Source: Int J Mol Sci. 2026 Aug 7;27(16):7089. doi: 10.3390/ijms27167089 (PMC13513820; doi:10.3390/ijms27167089)
Supplement: Supplementary file 1 [file ijms-27-07089-s001.zip › ijms-4427161-supplementary.pdf]

Supplementary Materials

ERβ-Score: An Interpretable Machine Learning–Based Scoring Function and Web Server for Estrogen Receptor β–Guided Drug Discovery in Triple-Negative Breast Cancer

Abbas Khan[1], Muhammad Ammar Zahid[1], Walid Kouidri[1], Osama Aboubakr Mohamed[1], Ahmed Mohammad Gharaibeh[1], Ladun Ibrahim Mohamed[1], Amani Anwar Al-Mansori[1], Mohamed Haitham Elsayed[1], Anwar Mohammad[2], Ameera Al-Jabiry[3], Mohanad Shkoor[3], Raed M. Al-Zoubi[4,5,6*], Abdelali Agouni[1*]

1. Department of Pharmaceutical Sciences, College of Pharmacy, QU Health, Qatar University, P.O. Box 2713, Doha, Qatar
2. Precision Health Analysis Unit, Translational Research, Dasman Diabetes Institute, Dasman, Kuwait
3. Department of Chemistry and Earth Sciences, College of Arts and Science, Qatar University, P.O. Box 2713, Doha, Qatar
4. Surgical Research Section, Department of Surgery, Hamad Medical Corporation, Doha, Qatar
5. Department of Biomedical Sciences, College of Health Sciences, QU Health, Qatar University, P.O. Box 2713, Doha, Qatar
6. Department of Chemistry, Jordan University of Science and Technology, P.O. Box 3030, Irbid, 22110, Jordan

Abbas Khan	email: abbas.khan@qu.edu.qa
Muhammad Ammar Zahid	email: mz1912625@qu.edu.qa
Walid Kouidri	email: wk1902655@qu.edu.qa
Osama Aboubakr Mohamed	email: oa2003649@qu.edu.qa
Ahmed Mohammad Gharaibeh	email: ag2107297@qu.edu.qa
Ladun Ibrahim Mohamed	email: lm2006178@qu.edu.qa
Amani Anwar Al-Mansori	email: aa2004409@qu.edu.qa
Mohamed Haitham Elsayed	email: me1901975@qu.edu.qa
Anwar Mohammad	email: anwar.mohammad@dasmaninstitute.org
Ameera Al-Jabiry	email: a.aljabiry@qu.edu.qa

Mohanad Shkoor email: mshkoor@qu.edu.qa

Raed M. Al-Zoubi email: ralzoubi@hamad.qa

Abdelali Agouni email: aagouni@qu.edu.qa

***Corresponding authors:**

- Prof. Abdelali Agouni, Department of Pharmaceutical Sciences, College of Pharmacy, QU Health, Qatar University, P.O. Box 2713, Doha, Qatar. **Email:** aagouni@qu.edu.qa
- Prof. Raed M. Al-Zoubi**,** Surgical Research Section, Department of Surgery, Hamad Medical Corporation, Doha, Qatar**. Email:** ralzoubi@hamad.qa

Table S1. The detailed parameters for each algorithm, including hyperparameters with search range.

Algorithm	Hyperparameter	Search Range
**RF / ET**	n_estimators	[100, 600], step 50
	max_depth	[3, 20]
	min_samples_split / min_samples_leaf	[2, 20] / [1, 10]
	max_features	[‘sqrt’, ‘log2’, 0.3, 0.5]
**XGBoost**	n_estimators / max_depth	[100, 600] step 50 / [3, 10]
	learning_rate	[10^{-3}, 0.3], log-uniform
	subsample / colsample_bytree	[0.5, 1.0]
	gamma / reg_alpha / reg_lambda	[0, 5] / [10^{-4}, 10] log / [10^{-4}, 10] log
**LightGBM**	n_estimators / max_depth / num_leaves	[100, 600] / [3, 12] / [20, 150]
	learning_rate / subsample / colsample	[10^{-3}, 0.3] log / [0.5, 1.0] / [0.5, 1.0]
	reg_alpha / reg_lambda	[10^{-4}, 10], log-uniform
**HistGradBoost**	max_iter / max_depth	[100, 500] step 50 / [3, 12]
	learning_rate / min_samples_leaf / l2_reg	[10^{-3}, 0.3] log / [5, 50] / [10^{-4}, 10] log
**GradBoost**	n_estimators / max_depth	[50, 400] step 50 / [2, 8]
	learning_rate / subsample / min_samples_split	[10^{-3}, 0.3] log / [0.5, 1.0] / [2, 20]
**SVM-RBF**	C / γ	[10^{-2}, 100] / [10^{-4}, 1.0], log-uniform
**MLP**	n_layers / units per layer	[1, 3] / {32, 64, 128, 256, 512}
	learning_rate_init / α (L2)	[10^{-4}, 0.1] / [10^{-5}, 0.1], log-uniform
**LogReg**	C / penalty	[10^{-3}, 100] log / {‘l1’, ‘l2’}
**kNN**	n_neighbors / weights / metric	[3, 30] / {uniform, distance} / {euclidean, manhattan, minkowski}

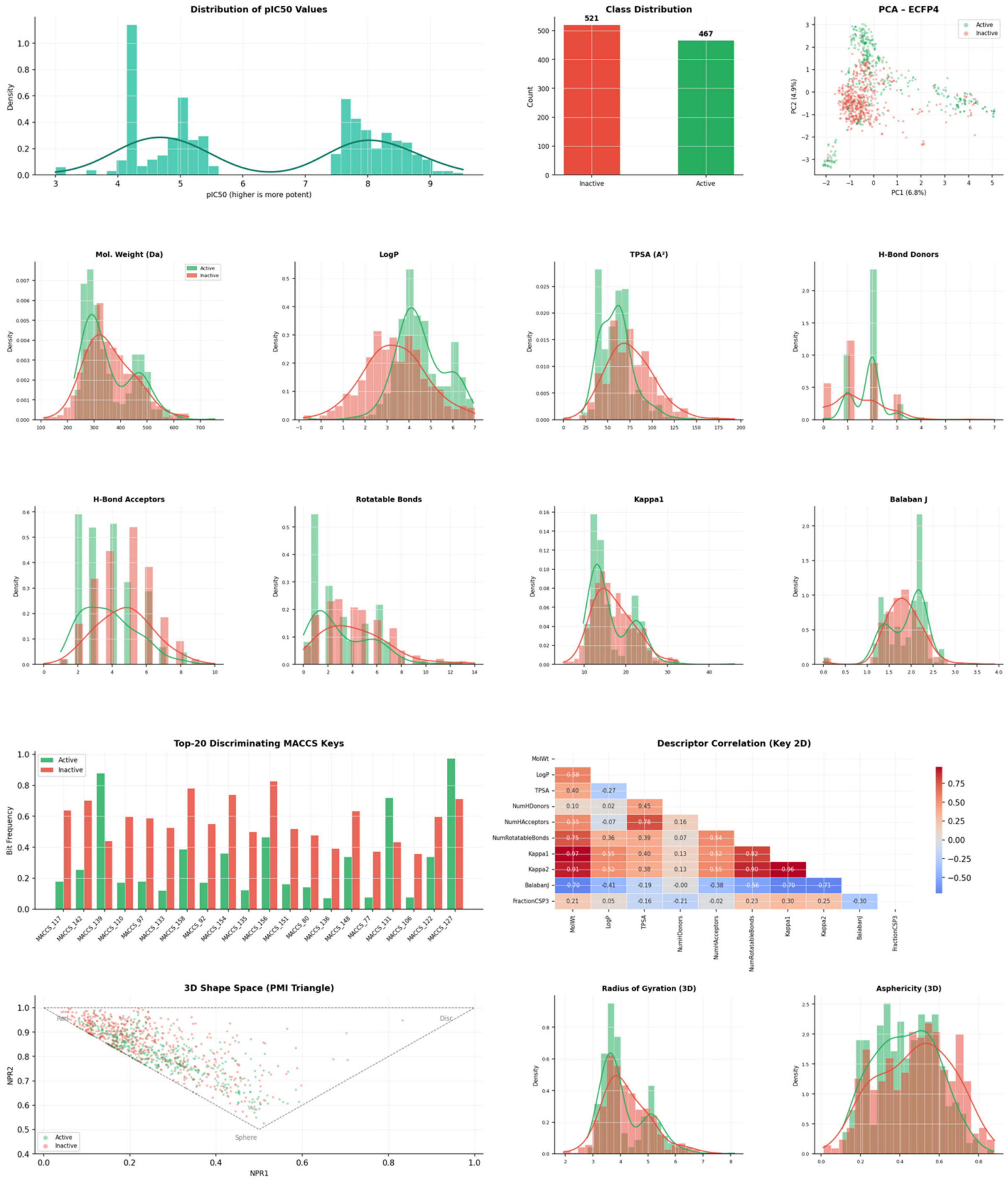

Figure S1. Fingerprint and descriptor characterization. Row 1: pIC50 distribution, class balance, ECFP4 PCA. Rows 2–3: Key 2D descriptor distributions by class with KDE overlays. Row 4: Top-20 discriminating MACCS keys, descriptor correlation heatmap. Row 5: 3D PMI triangle, radius of gyration, asphericity.

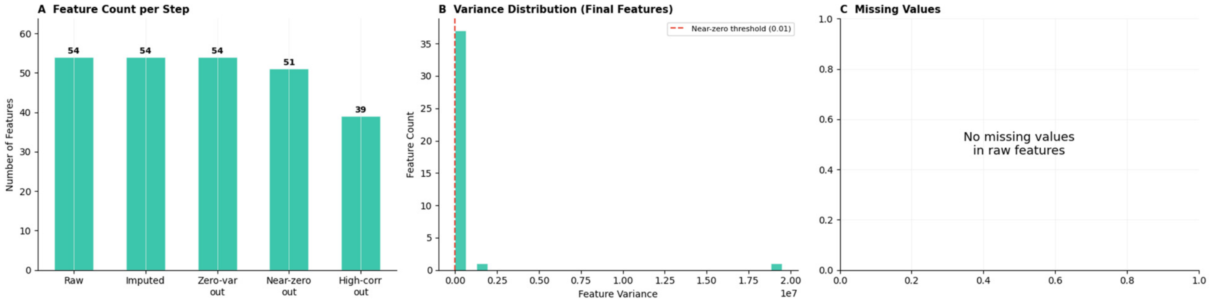

Figure S2. Feature preprocessing visualization. **(A)** Feature count per pipeline step. **(B)** Variance distribution of the final 39 features. **(C)** Missing-value audit (none detected).

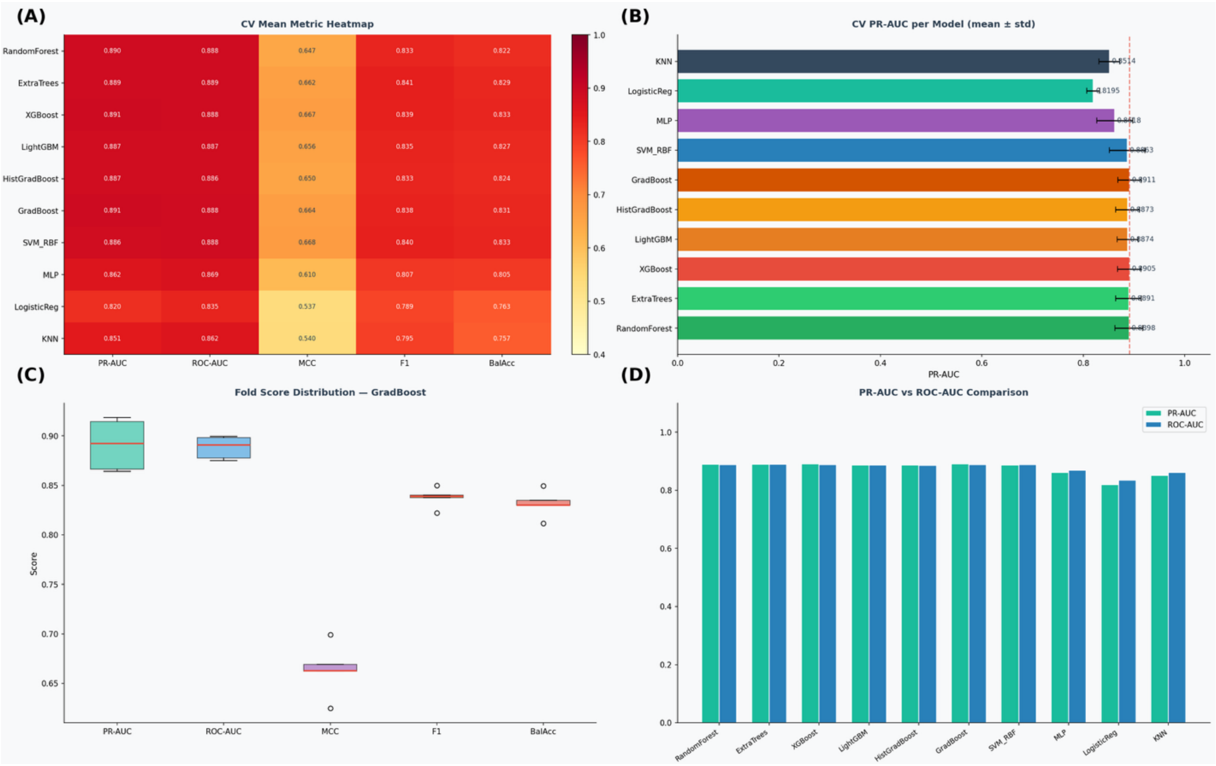

Figure S3. Cross-validation overview. **(A)** Metric heatmap across all models. **(B)** PR-AUC bar chart with std error. **(C)** Fold-level score distributions for GradBoost. **(D)** PR-AUC vs. ROC-AUC comparison.

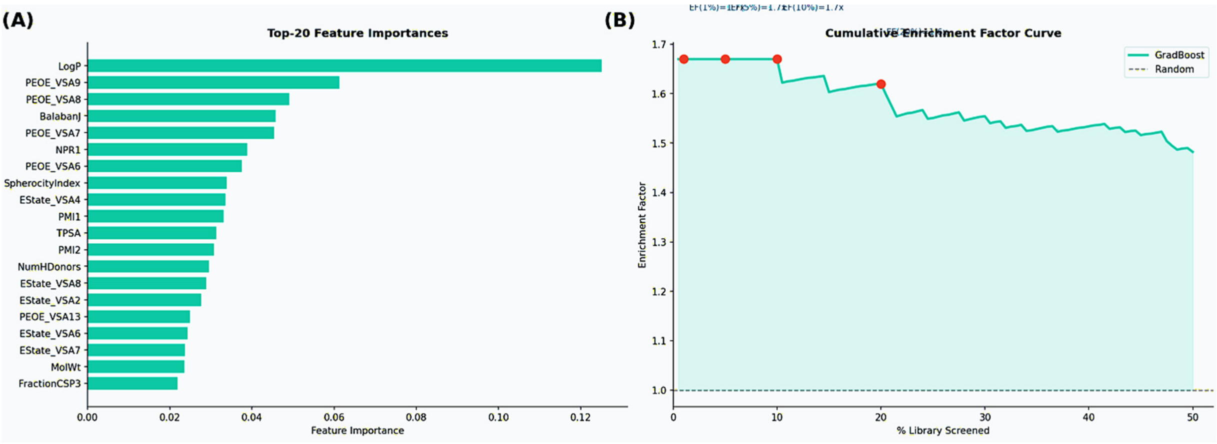

Figure S4: **(A)** Top 20 GradBoost feature importances. **(B)** Cumulative enrichment factor curve.

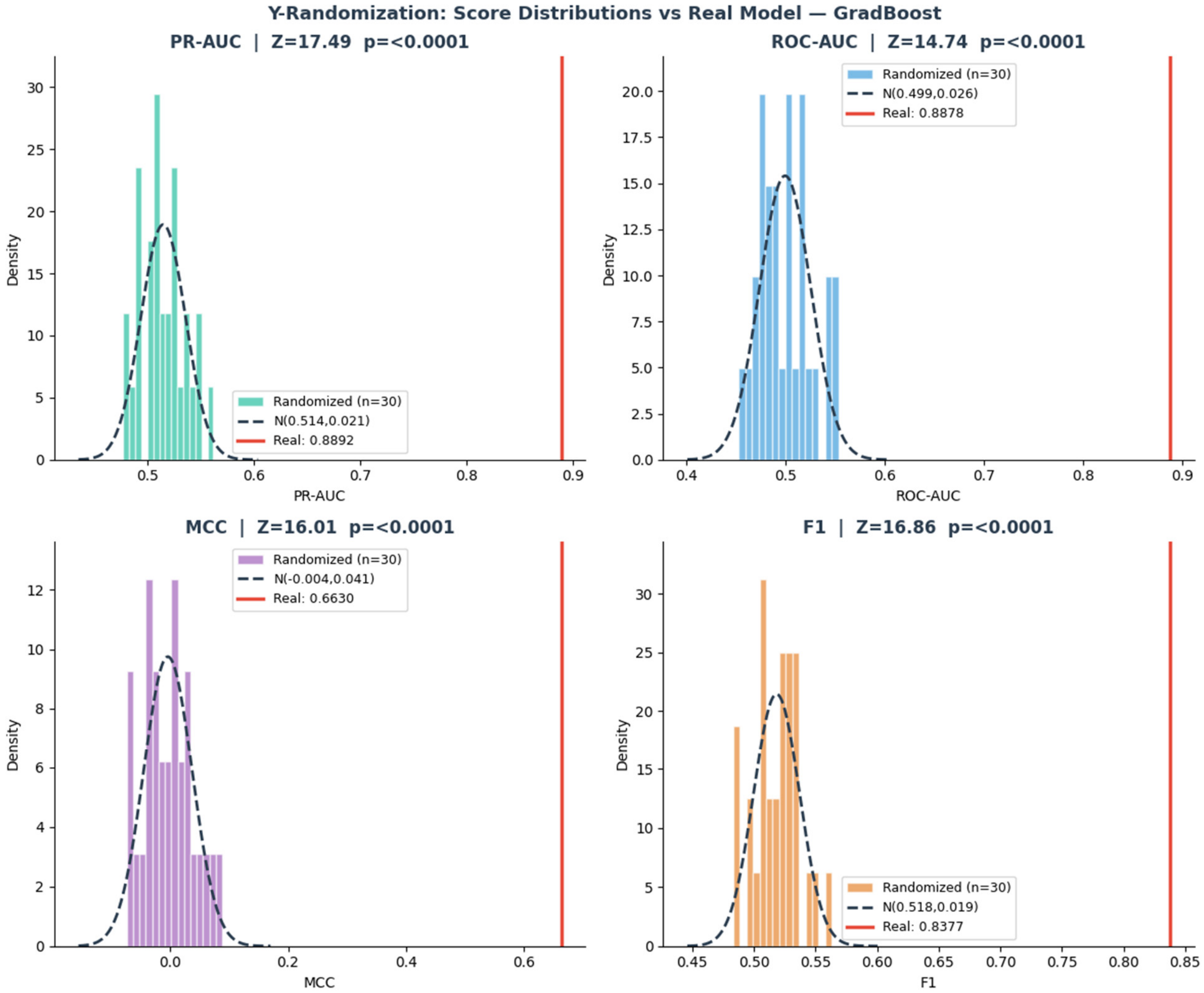

Figure S5. Y-randomization histogram overlays. Randomized distributions with fitted normal curves vs. real model values (red lines).

Table S2. External validation performance of ERβ-Score on an independent, non-ChEMBL Tox21 ERβ bioassay set (agonist assay PubChem AID 1259394; antagonist assay AID 1259378; n = 5,913 compounds), compared against the internal scaffold-holdout test reported in the main text.

Metric	Internal test (n = 339)	External Tox21 set (n = 5,913)
ROC-AUC	0.864	0.709 (95% CI 0.686–0.732)
MCC	0.578	0.124 (95% CI 0.085–0.165)
Balanced accuracy	0.795	0.538
Sensitivity (Active recall)	not separately reported *	0.099
Specificity (Inactive)	not separately reported *	0.976
Precision (PPV, Active)	not separately reported *	0.287

** The main text reports PR-AUC/ROC-AUC/MCC/F1/BalAcc for the internal test but not a raw confusion matrix; sensitivity/specificity are computed here directly from the external predictions.*

Table S3. Applicability-domain (AD) stratification of external validation performance (kNN-Tanimoto/ECFP4 gate, threshold $\tau = 0.2419$).

Subset	n	Active (n)	Sensitivity	Specificity	ROC-AUC
In-Domain (score $\geq \tau$)	2,052	286	0.105	0.976	0.735
Out-of-Domain	3,861	239	0.092	0.976	0.696

Table S4. Illustrative reference ERβ ligands present in the external Tox21 validation set, comparing the Tox21 consensus call with the ERβ-Score model prediction.

Compound	Tox21 call	Model prediction	P(Active)
17β-Estradiol	Active	Active	0.971
Zearalenone	Active	Active	0.921
Coumestrol	Active	Active	0.931
4-Hydroxytamoxifen	Active	Active	0.686
Genistein	Active	Inactive (missed)	0.350
Diethylstilbestrol	Active	Inactive (missed)	0.203
Fulvestrant	Active	Inactive (missed)	0.277
Estrone	Active	Inactive (missed)	0.384
